# A Pan‐TE Map Reveals the Important Role of Transposable Elements in Gene Expression and Phenotypic Diversity in 2,311 Rapeseed Accessions

**DOI:** 10.1002/advs.202512036

**Published:** 2025-12-08

**Authors:** Zhiquan Yang, Haiyan Fan, Yifan Chen, Jiawei Li, Minjian Chen, Yingying Tan, Liang Guo, Jintao Li, Kede Liu, Qing‐Yong Yang

**Affiliations:** ^1^ National Key Laboratory of Crop Genetic Improvement and Hubei Engineering Technology Research Center of Agricultural Big Data Huazhong Agricultural University Wuhan 430070 China; ^2^ Yazhouwan National Laboratory Sanya 572025 China; ^3^ College of Life Sciences Xinyang Normal University Xinyang 464000 China

**Keywords:** Brassica napus, multi‐omics, pan‐TE, TE insertion polymorphism, transposable element

## Abstract

Transposable elements (TEs) play an important role in shaping gene transcription regulatory networks and driving genome evolution. However, there is still little understanding for TE polymorphisms at the species level of rapeseed and their impacts on gene expression and phenotypic formation. This study systematically identified 8 268 255 TEs from 14 rapeseed genomes and constructed the first rapeseed pan‐TE map and then obtained TE insertion polymorphisms (TIP) in 2311 oilseed rape accessions (*Brassica napus*). Integrative analysis of transcriptome data suggests TEs modulate transcription by diverse *cis*‐regulatory mechanisms and 67% TEs related to nearby gene promote transcription. TE‐GWAS identified 1427 TEs in 80 loci associated with 15 traits. Further multi‐omics analysis identified a TE insertion in *BnaA03.FLCb*. suppresses its transcription and ultimately results in early flowering. Besides, one CACTA‐like insertion in *BnaA09.CYP78A9* reveals the regulatory mechanism how TE‐derived *cis*‐regulatory elements promote gene expression to regulate silique length. These results provide a TIP map at the species level and demonstrate that TEs play an important role in transcription regulatory and breeding improvement in rapeseed.

## Graphical abstract

This study presents the first pan‐transposable element (TE) atlas in rapeseed, profiling over 8 million TEs across 2311 accessions. Integrative analyses reveal that TEs broadly shape gene expression through diverse regulatory mechanisms, with key insertions in *BnaA03.FLC* and *BnaA09.CYP78A9* linked to flowering time and silique development. These findings provide fundamental insights into TE‐mediated regulation and valuable resources for molecular breeding.

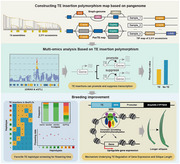

## 1 Introduction

Transposable elements (TEs) are mobile genetic sequences capable of replicating or relocating within host genomes, profoundly shaping genomic architecture and function.^[^ ^1^ ^]^ Based on their replication mechanism, TEs are categorized into two classes: ‌Class I retrotransposons‌ (which transpose via an RNA intermediate) and ‌Class II DNA transposons‌ (which move directly via a “cut‐and‐paste” mechanism).^[^ ^2^ ^]^ These classes are further subdivided into distinct subfamilies defined by structural features such as terminal repeats and transposase‐coding sequences.^[^ ^3^ ^]^ In most plants, TEs and their remnants account for a large proportion of the genome. However, in different plants, the coverages and proportions of different TE classes vary greatly.^[^ ^4^ ^]^ For a long time, TE was once referred to as “junk DNA”.^[^ ^5^ ^]^ But recent studies have shown that TEs play key roles in regulating gene expression, phenotype formation,^[^ ^6^ ^]^ and driving genome evolution.^[^ ^1^, ^4^, ^7^ ^]^ TEs can introduce *cis*‐regulatory elements (CREs) that influence gene expression levels, modify 3D chromatin architecture, and give rise to novel regulatory genes, including non‐coding RNAs and transcription factors (TFs).^[^ ^1^, ^4^, ^8^ ^]^ TE insertions are considered to be deleterious in nature considering that they threaten genome stability, yet their accumulation can promote phenotypic evolution by providing the raw material for genetic change.^[^ ^8^, ^9^ ^]^ Nevertheless, quantitative insights into how genome‐wide TEs influence gene regulatory networks and adaptive traits at the species level remain critically underexplored in plant genomes.

Rapeseed is an important oil crop and a major source of vegetable oil and protein in human production.^[^ ^10^ ^]^ As a young allotetraploid species, rapeseed (*Brassica napus*, 2*n* = 4*x* = 38, AACC) was originated from natural hybridization between the two ancestors species, *Brassica rapa* (*B. rapa*, 2*n* = 2*x* = 20, AA) and *Brassica oleracea* (*B. oleracea*, 2*n* = 2*x* = 18, CC), about 7500 years ago.^[^ ^11^ ^]^ During long‐term breeding and genetic improvement, rapeseed has undergone intensive selection for ‌ecological adaptation‌, ‌yield optimization‌, and ‌enhanced oil quality‌. Recent advances in long‐read sequencing technologies have enabled de novo sequencing and high‐quality assembly of multiple rapeseed genomes, laying the foundation for ‌pan‐genome construction‌ and genome‐wide identification of structural variations (SVs), including TE polymorphisms. TE insertions have been reported related to natural variations of key traits such as flowering time, self‐incompatibility, pod shattering, oil quality, glucosinolates, and yield.^[^ ^12^ ^]^ Some of the TEs act as enhancers to promote adjacent gene expression.^[^ ^12^, ^13^ ^]^ Therefore, a comprehensive evaluation of TE effects on gene expression and phenotype variation is necessary for understanding the mechanism of transcriptional regulatory and phenotype formation in rapeseed. However, there is no systematic identification of TE insertion polymorphisms in rapeseed genome at the species level. In particular, there lacks integrating multi‐omics data to explore how TEs regulate gene expression and resulting into phenotypic variation across the species.

In this study, we systematically identified and compared the distribution characteristics of TEs from 14 published rapeseed genomes and constructed the first rapeseed pan‐TE map. With this pan‐TE map, we identified TE insertion polymorphisms (TIPs) using resequencing data of 2311 accessions. We further explored genetic basis and regulatory mechanisms that TEs affect gene expression and phenotype by integrating population‐level multi‐omics data. In conclusion, these results can provide data support for mining favorable TE variations in rapeseed and exploring their regulatory mechanism for phenotypic formation, as well as a reference for the discovery of the effects of TEs in other plants.

## 2 Results

### 2.1 Construction of Rapeseed Pan‐Genome

**Figure 1:**
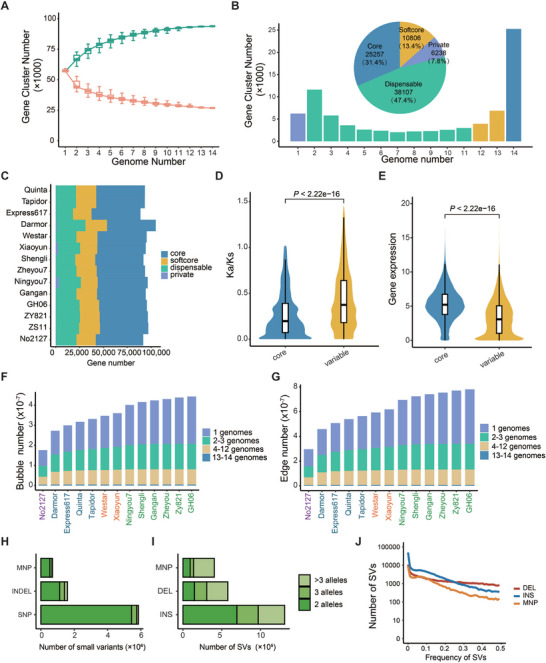
Construction of *Brassica napus* pangenome. A) *Brassica napus* pangenome at gene level. Orange and green boxes represent core and all gene clusters, respectively. B) Genome number of different gene clusters. Brown, yellow, blue, and purple bars represent core, softcore, dispensable, and private gene clusters, respectively. C) Gene number of different gene clusters in 14 genomes. D,E) *K*_a_/*K*_s_ values (D) and expression levels (E) of core and variable gene clusters. Blue and yellow bars represent core (*n* = 47 736) and variable gene clusters (*n* = 37 558). *p*‐values were calculated using a two‐tailed Wilcoxon signed‐rank test. F,G) Numbers of bubbles (F) and edges (G) in *B. napus* pangenome based on 14 genome assemblies. Bars with different colors represent bubbles and edges present in 1 (purple), 2–3 (blue), 4–12 (yellow), and 13–14 (brown) genomes. Texts of *x* axis with different colors represent synthesized (purple), winter (blue), semi‐winter (green), and spring (orange) accessions. H,I) Numbers of different kinds of small variations (H) and structural variations (SVs) (I). Bars from light to deep green represent variations with two (light green), three (median green), and more than three (deep green) alleles. J) Frequency distribution of SVs. The number of rapeseed accessions is 2311. Lines with different colors represent deletions (red), insertions (blue), and multiple nucleotide polymorphisms (MNPs) (orange).

To construct a comprehensive pan‐genome reflecting the genetic diversity of rapeseed (*Brassica napus*), we systematically integrated genome assemblies and annotations from 14 representative accessions, including four winter‐type, three spring‐type (including a re‐synthetic rapeseed accession, No2127), seven semi‐winter‐type oilseed rape accessions (Table S1, Supporting Information). A total of 1 461 500 genes were annotated from the 14 genome assemblies. These genes were classified into 93 776 gene clusters based on sequence similarity of protein‐encoding genes, including 25 257 core (*n* = 14), 10 806 softcore (*n* = 12–13), 38 107 dispensable (*n* = 2–11), and 6238 private (*n* = 1) clusters according to their frequency in the 14 genome assemblies.^[^ ^14^ ^]^ Thereinto, the core gene clusters account for the largest proportion (**Figure** **1**A,B), while private gene clusters contained the least proportions of protein‐coding genes in each accession and are linage‐specific (Figure 1B,C). Compared with than variable (softcore, dispensable, and private) genes, core genes showed higher levels of expression and experienced stronger purification selection pressure (lower *K*_a_/*K*_s_) (Figure 1D,E). Functional enrichment analysis further demonstrated that core genes were predominantly associated with fundamental biological processes, such as photosynthesis and electron transport chain (Figure S1, Supporting Information). Private genes were enriched in biological processes related to the response to stress, such as trichome development and regulation of cellular response to heat, which may render rapeseed varieties different response to environmental changes (Figure S1, Supporting Information).

To comprehensively represent the genetic diversity across 14 accessions, we next constructed a rapeseed pangenome. In a typical pangenome reference, genomic data from a population can be organized into an edge‐based sequence variation graph. We applied the Minigraph‐Cactus pipeline to construct a base‐level variation graph anchored to the ZS11 reference genome, incorporating structural variant (SV) calls from the BnIR database,^[^ ^15^ ^]^ in which 14 genome assemblies can be represented as different paths composed of sequence nodes. Finally, we constructed a rapeseed graph with a length of 1.68 Gb (measured as the sum of all nodes). There were a total of 922 Mb of non‐reference sequence distributed across 7 687 482 bubbles, encompassing 8 143 465 small variants (<50 bp) and 229 226 SVs (≥50 bp), and the resynthesized rapeseed line No2127 contributed the most nonreference sequence (Figure 1F–I).

We further constructed a population‐scale SV map by genotyping 2311 resequenced rapeseed accessions against the graph pangenome using PanGenie.^[^ ^16^ ^]^ Frequency distribution analysis showed that 55.7% of SVs occur at low frequencies (MAF <5%) and the number of insertions (INS), deletions (DEL), and multiple nucleotide polymorphisms (MNP) gradually decreases as their frequency increased (Figure 1J). This distribution pattern is similar to previous research results,^[^ ^17^ ^]^ suggesting that INS and DEL may be under stronger selection pressure or eliminated from the genome by purifying selection owing to their large impact on genome at the sequence scale.^[^ ^18^ ^]^

### 2.2 Construction of Rapeseed Pan‐TE Map

**Figure 2:**
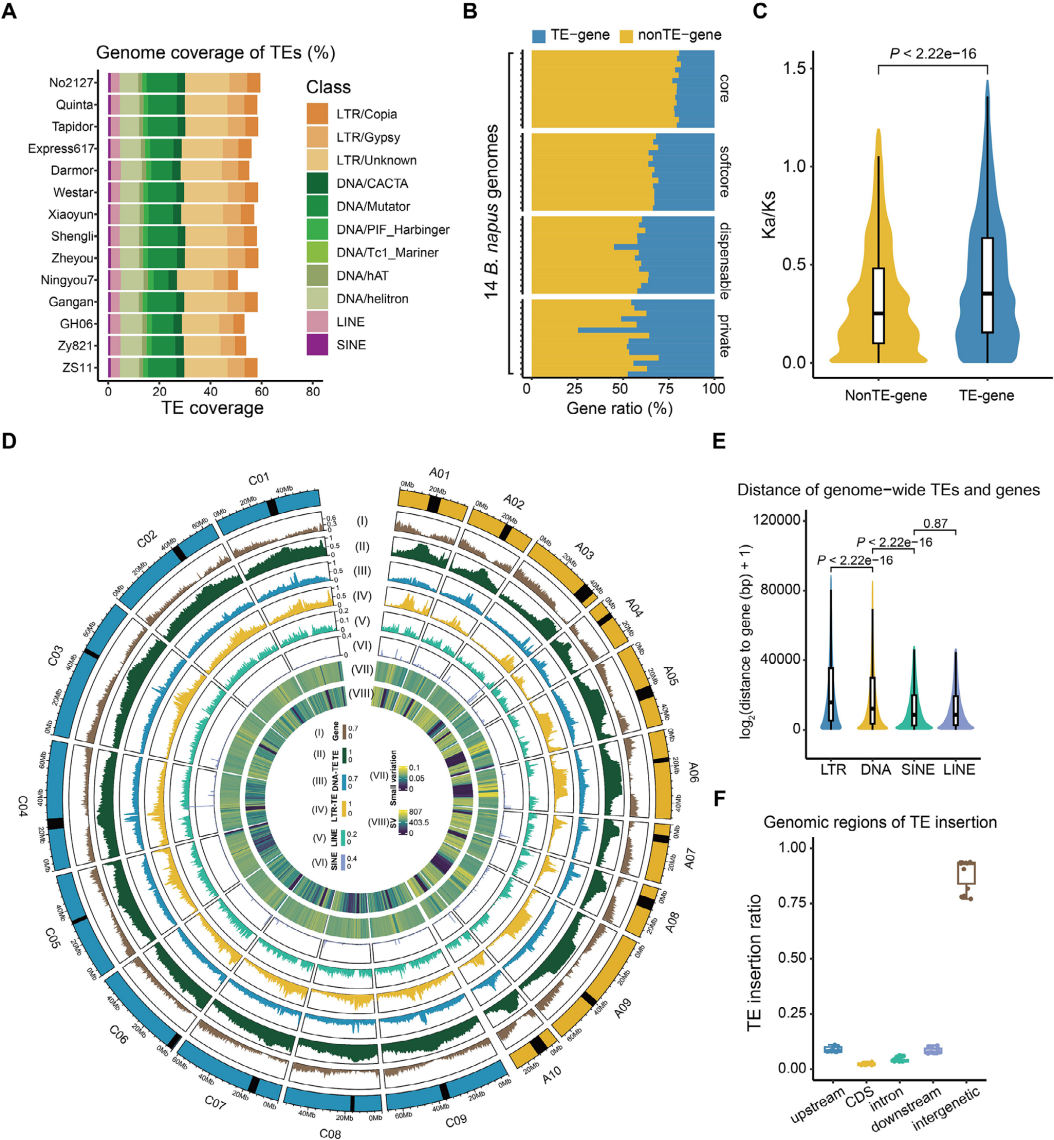
Analysis of transposable elements (TEs) in 14 *Brassica napus* genomes. A) Genome coverage of TEs in 14 *B. napus* genomes. Bars with different colors represent TEs with different classes or subclasses. B) Ratios of genes in different gene clusters with TE insertions and no TE insertions. C) *K*_a_ */K*_s_ values of genes with TE insertions and no TE insertions. Bars with different colors represent genes with TE insertions (blue, *n* = 18 967) and no TE insertions (yellow, *n* = 49 146). *p*‐values were calculated based on the two‐tailed Wilcoxon rank‐sum test. D) Genomic distribution feature of TEs. Different tracks (I–VIII) indicate the densities of genes, TEs, DNA‐TEs, LTR‐TEs, LINE‐TEs, and SINE‐TEs, small variations (I–VII) and abundance of structural variations (SVs) (VIII). E) Distance between TEs and adjacent genes. Blue, yellow, green, purple, and brown violin plots represent LTR‐TEs (*n* = 210 743), DNA‐TEs (*n* = 490 316), SINE‐TEs (*n* = 33 170), and LINE‐TEs (*n* = 39 937), respectively. *p*‐values were calculated based on the two‐tailed Wilcoxon rank‐sum test. F) Genomic distribution of TE insertions. Blue, yellow, green, purple, and brown boxes and points represent TE insertions in upstream, CDS, intron, and downstream of genes and intergenic regions, respectively. In (C) and (E), the two‐sided Wilcoxon rank‐sum tests were performed, and the *p*‐value is indicated, respectively.

A total of 8 268 255 transposable elements (TEs) were systematically annotated across 14 rapeseed genomes using RepeatMasker,^[^ ^19^ ^]^ with an average genomic coverage of 323.76 Mb per genome (**Figure** **2**A and Table S2, Supporting Information). TE coverage exhibited substantial inter‐genomic variation, ranging from 56.47% in the Ningyou7 genome to 62.10% in the No2127 genome (Figure 2A). Despite this variability, the proportional composition of major TE classes remained conserved among accessions. Among Class I elements, long terminal repeat (LTR) retrotransposons dominated, accounting for 23.77–29.42% of genomic coverage, while the two main non‐LTR retrotransposons, long interspersed nuclear elements (LINEs), and short interspersed nuclear elements cover 3.41–4.07% and 0.82–1.13%, respectively. Class II elements were predominantly represented by terminal inverted repeat (TIR) transposons (13.11–18.35%), while non‐TIR elements (8.93–10.76%) consisted primarily of Helitrons (7.72–8.87%). When comparing ratios of genes with TE insertions for core, softcore, dispensable, and private gene clusters, we found that core genes had the lowest proportion of TE insertions, followed by softcore, dispensable, and private genes, further suggesting that core genes retain fewer variations (Figure 2B), consistent with enhanced purifying selection pressure acting on these genes without TE insertions^[^ ^8^ ^]^ (*p*‐value <2.22 × 10^−6^, Wilcoxon rank‐sum test, Figure 2C). A comprehensive pan‐transposable element (pan‐TE) library consisting of 15 369 TE families was constructed based on TEs in 14 rapeseed genomes using the panEDTA pipeline.

In plants, TE insertions are usually family specific, which lead to distinct distributions in the genome.^[^ ^20^ ^]^ TE distribution in rapeseed genome confirmed the negative correlation between TE density and gene density in plant genomes^[^ ^17^, ^21^ ^]^ (Figure 2D, Figure S2, Supporting Information). Notably, distribution patterns of Class I/II TEs showed inverse correlations with SINE/LINE elements, with the latter exhibiting pronounced enrichment in genic proximal regions compared to LTR‐TEs and DNA‐TEs (Figure 2E, Figure S2, Supporting Information). These results indicated that the distribution of SINEs and LINEs is distinct from that of Class I and II TEs. Permutation tests statistically validated the non‐random genomic distribution of TEs (*p*‐value <0.001, Figure 2F and Figure S3, Supporting Information).

To assess the evolutionary role of TE insertions in rapeseed, we calculated the insertion time of 111 330 intact LTR‐TEs across these genomes (Figure S4, Supporting Information), and explored the correlation between them and recent genome evolution events including separation of *Brassica rapa* (*B. rapa*) and *Brassica oleracea* (*B. oleracea*, ≈4.6 million years ago)^[^ ^22^ ^]^ and rapeseed formation (≈7500 years ago).^[^ ^11^, ^22^ ^]^ Comparative genomic analysis revealed that the vast majority of LTR‐TE insertions in both subgenomes postdated the speciation event between *B. rapa* and *B. oleracea*. The A subgenome of *B. napus* (A_n_) exhibited a significantly higher proportion of recent insertions. The 92% of intact LTRs in the A_n_ subgenome were accumulated within the last one million years, compared to 65% in the C_n_ subgenome, and higher LTR element were amplified in the A_n_ subgenome compared to the C_n_ subgenome after rapeseed formation. Furthermore, the C_n_ subgenome displayed one outbreak peak of Copia‐LTR elements at around 1.2 million years ago, which closely mirrors the observed divergence between the diploid progenitor genomes (*B. rapa* and *B. oleracea*).^[^ ^12^, ^23^ ^]^

To investigate TE insertion polymorphisms (TIP) in 2311 *Brassica napus* accessions, we first constructed a pan‐TE map based on the presence or absence of the same TE in different alleles in pangenome. Next, we obtained the genotypes of TEs by mapping the resequencing reads of 2311 accessions to the pan‐TE map. We found 85.14% of TEs exit in all accessions, suggesting that most TEs in *B. napus* are conserved. Only 14.86% of TEs showed polymorphisms among accessions, with most polymorphic TEs having low frequencies (Figure S5, Supporting Information).

### 2.3 The Impact of TE Insertions on Gene Expression

**Figure 3:**
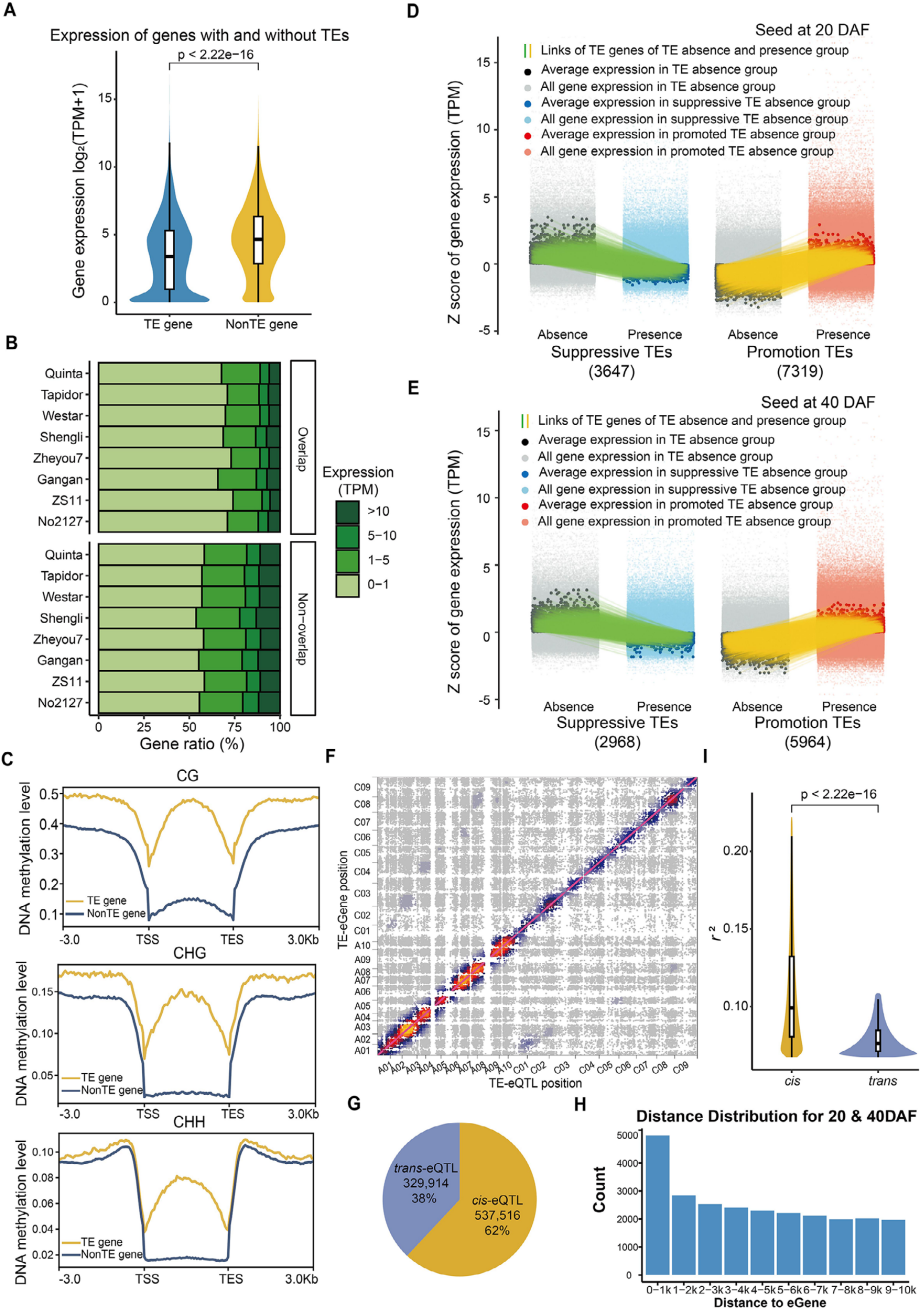
Analysis about the impact of transposable element (TE) insertions on gene expression. A) Maximum expression levels in different tissues of genes with (*n* = 28 804) and without transposable elements (TEs) (*n* = 72 194). *p*‐value was calculated based on the two‐tailed Wilcoxon rank‐sum test. B) Summary of expression levels in leaves of genes with and without TEs. Light to dark green bars represent gene expression levels (TPM) of 0–1, 1–5, 5–10, and more than 10. C) DNA methylation levels of genes with and without TEs. Top, median, and bottom panels show average methylation levels under CG, CHG, and CHH contexts. Blue and yellow lines represent DNA methylation levels of genes with and without TEs, respectively. D,E) Effect of TEs on the expression of associated genes (TE genes) based on population‐level gene expression datasets of seeds at 20 DAF (D) and 40 DAF (E). Gene expression levels for the absence and presence genotype groups (of corresponding TE). The *x* axis shows two groups associated with suppression and promotion TEs, respectively. The *y* axis shows the normalized (*z*‐score) expression values. The green/yellow lines link the average expression values from each gene for their presence and absence of genotype groups. F,H) Summary of TE‐eQTL mapping. F) Global heatmap between lead TEs and eGenes of TE‐eQTLs. The *x* and *y* axes show genomic positions of TE‐eQTLs and eGenes, respectively. Yellow to gray dots represent density of TE‐eQTLs. G) Numbers and ratios of *cis*‐ and *trans*‐eQTLs. H) Distances to eGenes of lead TEs. I) Gene expression variance explained by *cis*‐ (yellow, *n* = 537 516) and *trans*‐eQTLs (blue, *n* = 329 914). *p*‐value was calculated based on the two‐tailed Wilcoxon rank‐sum test.

TEs can interact with host genes, resulting in altered gene expression and regulatory networks.^[^ ^24^ ^]^ To investigate the impact of TEs on gene expression in the rapeseed genome, we collected the RNA‐seq data from leaves of eight accessions to compare the expression levels of 223 045 and 550 020 genes with and without TE insertions in their gene bodies,^[^ ^12^ ^]^ respectively. The genes with TE insertions show significantly lower expression levels than those without TE insertions (*p*‐value <2.22 × 10^−6^, Wilcoxon rank‐sum test, **Figure** **3**A,B), which is similar to those in other plants,^[^ ^6^ ^]^ indicating that TE insertions affected transcription. We further compared DNA methylation in genes with and without TE insertions using the published whole genome bisulfite sequencing (WGBS‐seq) dataset from two accessions (B409 and 2063A) at different tissues and development stages.^[^ ^25^ ^]^ Genes with TE insertions showed significantly higher methylation levels (Figure 3C), which may potentially lead to lower expression levels. Considering the genes without TE insertions experienced strong negative selection, we deduced that their high DNA methylation and low expression levels may be related to genomic evolution.^[^ ^8^ ^]^ To more finely explore how TE insertions impact on the expression levels of adjacent genes, we collected published population‐level gene expression datasets from seeds of 309 accessions at 20 DAF (Days After Flowering) and 40 DAF,^[^ ^26^ ^]^ and obtained a total of 55 465 expressed genes. Based on the approach used in previous studies, each TE was then assigned to its closest gene to observe impact on the expression levels of adjacent genes.^[^ ^27^, ^28^ ^]^ Totally, 72% (25 627) of TEs showed no detectable effect on adjacent gene expression (Figure S6, Supporting Information). The remaining TEs were categorized as promotive or suppressive based on whether they significantly upregulated or downregulated the expression of adjacent genes. In total, 66.74–66.77% of TEs exhibited promotive effects (7319 of 10 966 at 20 DAF; 5964 of 8932 at 40 DAF), nearly double the frequency of suppressive TEs (3647 at 20 DAF; 2968 at 40 DAF) (Figure 3D,E). Moreover, the proportions of promoted and suppressed TEs showed no significant difference between the two orientations (Figure S7, Supporting Information). Among the 9666 TEs with regulatory influence, 3107 consistently promoted and 1147 consistently suppressed adjacent gene expression levels across both two developmental stages (Figure S6, Supporting Information), supporting the stability of TE‐mediated regulatory effects. And based on the summary of different distances of TE insertion upstream and downstream of the gene, similar trends were all presented. Furthermore, the statistical analysis of TE insertions at different distances from the upstream and downstream regions of genes show the similar ratios (Figure S8, Supporting Information). These results indicate that the promotive effect of TEs can be stable across tissues.

**Figure 4:**
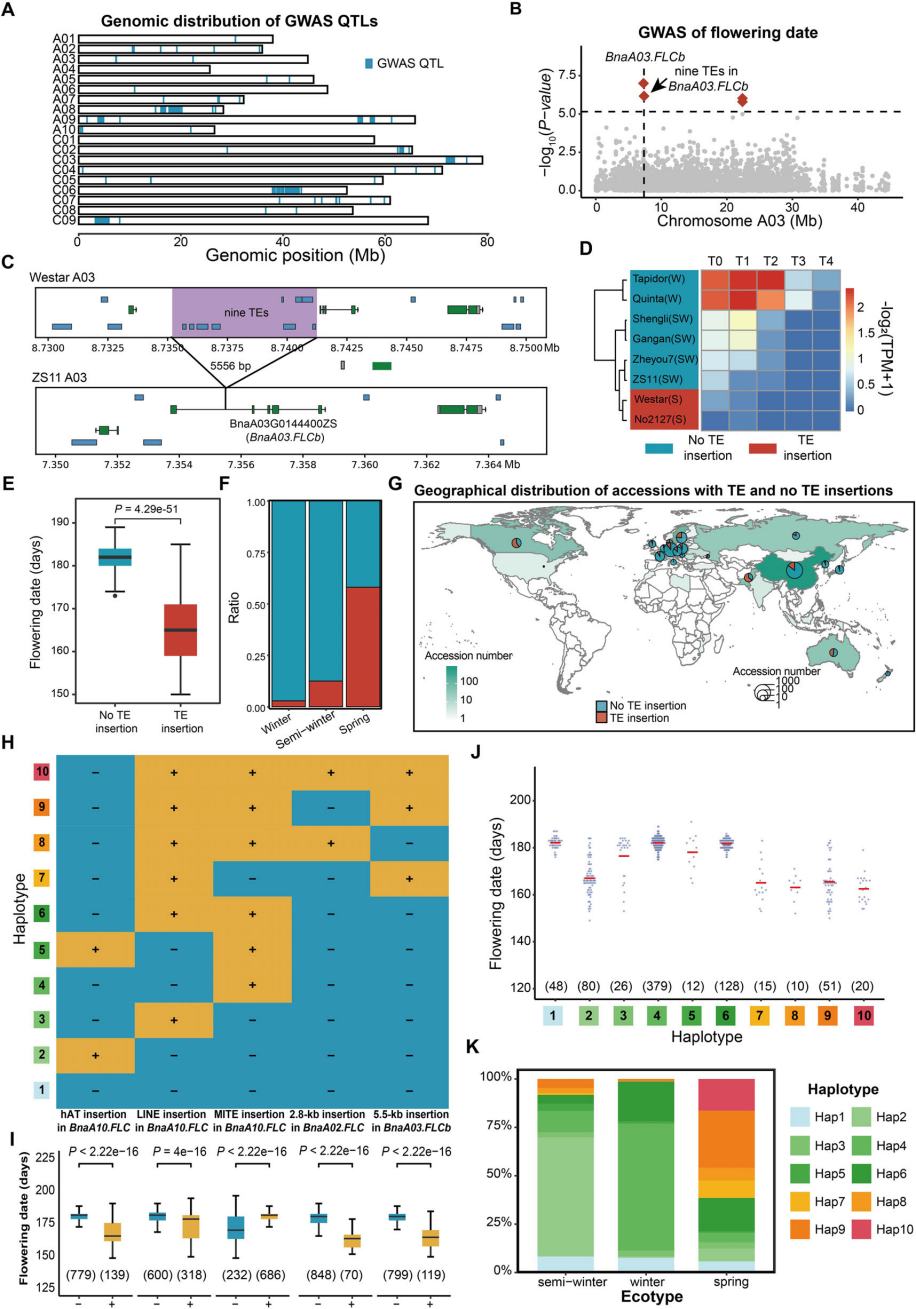
**Comparison of transposable element (TE) insertions in three ecotypes of *Brassica napus***. A) Genomic distribution of GWAS loci. Blue regions represent GWAS loci associated with 15 phenotypes. B) Local GWAS Manhattan plot of flowering time based on TE genotypes. The red diamonds represent significant TEs associated with flowering time. The cutoff of GWAS is −log_10_ (1/*n*), *n* = 148 323. C) Schematic diagram of one 5556 bp insertion containing nine TEs in the intron of *BnaA03.FLCb*. D–F). Flowering dates (D), gene expression levels (E) and geographical distribution (F) of accessions with and without TE insertions. Blue and red boxes or pies represent accessions with and without TE insertions, respectively. In (E), blue to red regions represent low (*n* = 593) to high (*n* = 117) gene expression levels. *p*‐value was calculated based on the two‐tailed Wilcoxon rank‐sum test. G) Phylogenetic tree of 2311 *B. napus* accessions. Branches with different colors represent spring (orange), winter (blue), and semi‐winter (green) accessions. Blue and red regions represent accessions with and without TE insertions, respectively. H) Haplotype combinations based on five insertions in 2311 accessions. I) Flowering dates of accessions with and without insertions. Yellow and blue boxes represent accessions with and without insertions, respectively. All *p*‐values were calculated based on the two‐tailed Wilcoxon rank‐sum tests. The numbers under the boxes indicate numbers of accessions with different haplotypes. J) Flowering periods of materials corresponding to 10 haplotype combinations. Purple dots represent flowering dates of different accessions, and red horizontal lines indicate the average flowering dates for each haplotype. The numbers under the points indicate numbers of accessions with different haplotypes. K) Proportions of different haplotype combinations across three ecotypes. Ratios of spring, winter, and semi‐winter accessions with and without TE insertions. Segments with 10 colors represent 10 haplotypes.

To further analyze the genome‐wide influence of TEs on transcriptions, we performed expression quantitative trait locus (eQTL) analysis integrating TE genotypes with transcriptome data.‌ Totally, 867 430 TE‐eQTLs were detected to be associated with expression levels of 42 111 genes (**Figure** **4**F, Table S3, Supporting Information), including 537 516 (61.97%) *cis*‐eQTLs (the distances between TEs and their regulated genes within 1 Mb) and 329 914 (38.03%) *trans*‐eQTLs, suggesting that TEs mainly *cis*‐regulate gene expression levels (Figure 3G). The number of *cis*‐eQTLs decrease as the distances between TEs and their target genes increase, further suggesting that TEs primarily regulate the expression of nearby genes (Figure 3H). Next, we compared the impact of TEs in *cis*‐eQTLs (*cis*‐TEs) and *trans*‐eQTLs (*trans*‐TEs) on gene expression, and found that the effects of *cis*‐TEs were significantly stronger than those of *trans*‐TEs (Figure 3I), indicating that *cis*‐TEs play more important roles on gene expression levels, which is similar to those found by eQTLs based on single nucleotide polymorphisms (SNPs) in rapeseed and other plants.^[^ ^29^, ^30^ ^]^

### 2.4 The Impact of TE Insertions on Phenotype

To explore how TE insertions influence phenotypes, we first performed TE‐GWAS for 18 traits from 502 accessions using published phenotypic datasets from BnIR database^[^ ^15^ ^]^ (Figure S9, Supporting Information). Totally, 1427 TEs in 80 loci were identified to be significantly associated with 15 traits, involving 3095 candidate genes (Figure 4A, Table S4, Supporting Information), which provide important data source for further genetic breeding. For example, as for seed traits, multiple TE insertions in *BnaMYB*s were identified to be associated with seed glucosinolate content, and a 3.5‐kb CACTA‐like TE in the upstream of *BnaA09.CYP78A9* (BnaA09G0560100ZS) was associated with seed weight (Table S4, Supporting Information). Those results were similar to those found in the previous studies.^[^ ^13^, ^17^ ^]^ Among them, a 5.5‐kb genomic fragment containing nine TEs inserted in the first intron of *BnaA03.FLCb* (BnaA03G0144400ZS) was found to be significantly associated with flowering time (Figure 4B,C). In the 14 assembled genomes, no 5.5‐kb TE insertion exist in *BnaA03.FLCb* of Westar and No2127 while other 12 genomes were with the 5.5‐kb insertion. Transcriptome analysis with eight rapeseed accessions with RNA‐seq data obtained from leaves at four development stages showed that the 5.5‐kb TE insertion suppressed the expression of *BnaA03.FLCb* in two spring accessions (Westar and No2127) and ultimately resulting in early flowering in *Brassica napus* (Figure 4E). Further epigenome datasets indicate that the 5.5‐kb TE insertion may recruit H3K27me3 and other heterochromatin signals, resulting in lower expression level of *BnaA03.FLCb*. In the 2311 *Brassica napus* accessions, the 5.5‐kb TE insertion primarily occurred in the spring rapeseed accessions (205 out of 355 accessions). And geographically, as core production regions for spring rapeseeds, Canadian and Australian accessions show higher frequencies of this insertion (Figure 4E–G, Table S5, Supporting Information). These results suggest that this TE insertion may be associated with ecotype differentiation and occurred during the spread of winter rapeseed to spring rapeseed production areas.

Given that previous studies have reported multiple SVs in *BnaFLC*s genes associated with flowering time including hAT, LINE, and MITE insertions in *BnaA10.FLC* and one 824‐bp insertion in *BnaA02.FLC*,^[^ ^12^, ^31^ ^]^ these variations may collectively contribute to the formation of three ecotypes. To elucidate how these SVs collectively modulate flowering time across ecotypes, we conducted haplotype combination analyses incorporating these known variations along with a novel 5.5‐kb insertion in *BnaA03.FLCb* identified here. Among 920 accessions used in GWAS,^[^ ^31^, ^32^ ^]^ 10 haplotype combinations with frequencies of more than 0.05 were retained (Figure 4H, Table S6, Supporting Information). Since most of these TE insertions and SVs were associated with earlier flowering except for the MITE insertion in *BnaA10.FLC* (Figure 4I), accessions carrying more *BnaFLC*s with variations generally exhibited earlier flowering among 10 haplotypes (Figure 4J). For instance, accessions with Hap7‐10 showed significantly earlier flowering time compared to other haplotypes (Figure 4J). Notably, accessions with Hap2 show similar flowering time (167 days of average flowing time) with Hap7‐10 (165, 160, 166, and 161 days of average flowing time, respectively) despite only containing the hAT insertion in upstream of *BnaA10.FLC* suggesting its strong effect on flowering regulation (Figure 4J). Ecotype‐specific haplotype distribution patterns were observed based on frequency analysis (Figure 4K). For winter and semi‐winter ecotypes, Hap4 and Hap2, respectively, displayed ecotype specificity, suggesting that MITE and hAT insertions in *BnaA10.FLC* determined their distinct flowering times (Figure 4K). As for spring ecotype, 104 (78%) of 132 spring accessions carried two variations and 68 spring accessions (51%) harbored three variations, demonstrating synergistic effects of these events in promoting early flowering (Figure 4K). Hap9 with two TE insertions in *BnaA10.FLC* and TE insertion of *BnaA03.FLCb−* in this study showed the highest frequency in spring rapeseed accessions (Figure 4K). In summary, during ecotype differentiation in *Brassica napus*, a series of TEs in different *BnaFLC*s may function synergistically to influence flowering time, ultimately contributing to the formation of distinct ecotypes.

### 2.5 An CACTA‐Like Insertion as an Enhancer Increases Silique Length and Seed Weight

**Figure 5:**
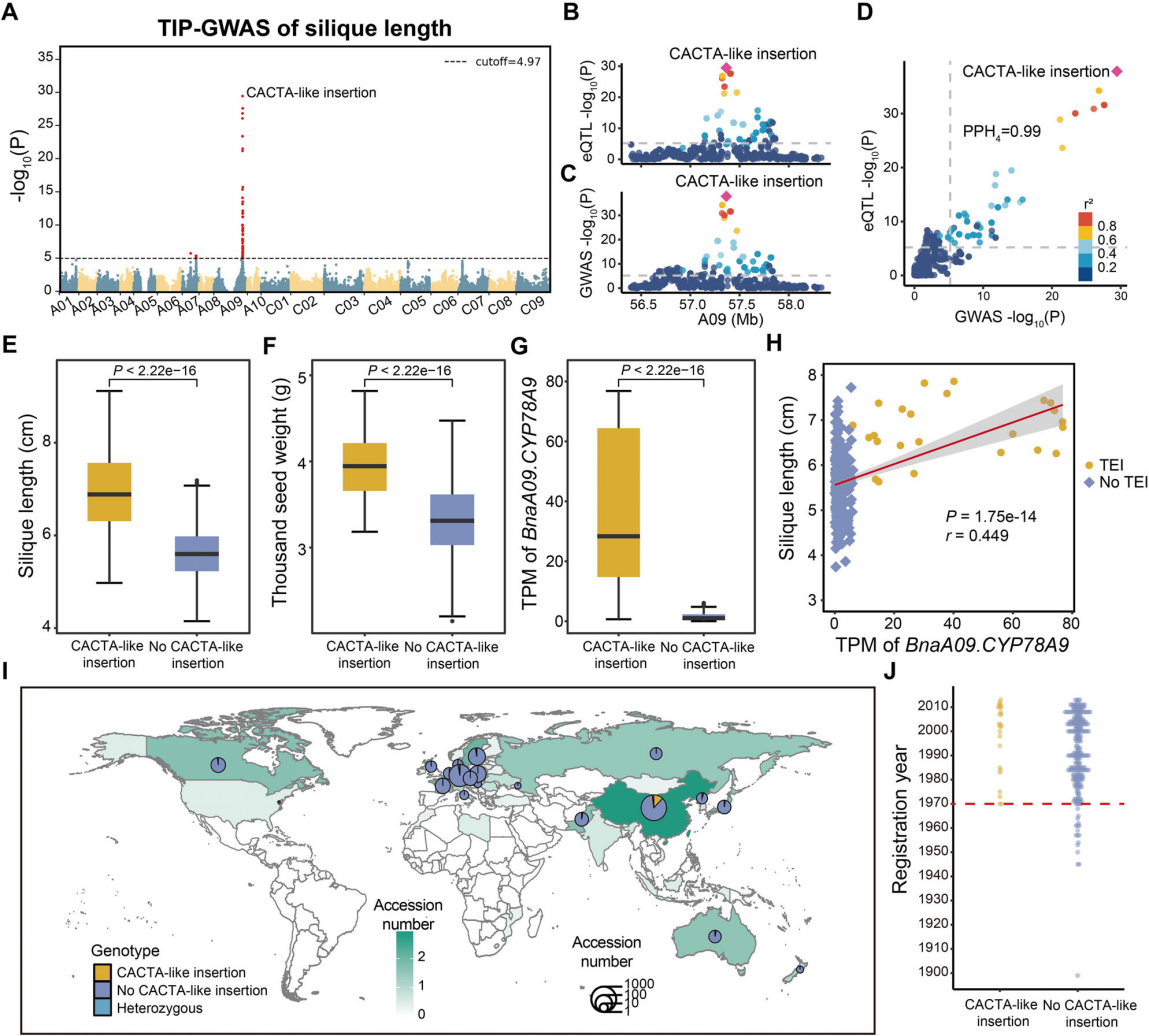
Analysis of one CACTA‐like insertion associated with yield. A) Global GWAS Manhattan plot of silique length based on transposable element (TE) genotypes. B,C) Local eQTL (B) and GWAS (C) Manhattan plots. D) Colocalization analysis of eQTL for *BnaA09.CYP78A9* and GWAS for silique length. E–G) Silique length (E), thousand‐seed weight (F), gene expression levels (G) of accessions containing the CACTA‐like insertion and no CACTA‐like insertion. Boxes with different colors represent accessions with the CACTA insertion (yellow, *n* = 60) and no CACTA‐like insertion (purple, *n* = 433). H) Relationship between gene expression levels of *BnaA09.CYP78A9* and silique length in populations. Dots with different colors represent accessions with the CACTA‐like insertion (yellow dots) and no CACTA‐like insertion (purple diamonds). I) Geographical distribution of 2311 accessions containing the CACTA‐like insertion and no CACTA‐like insertion. Yellow, purple and blue pies represent accessions with, without TE insertions and heterozygous genotype, respectively. J) Registration year of 991 accessions containing the CACTA‐like insertion and no CACTA‐like insertion. Dots with different colors represent accessions with the CACTA‐like insertion (yellow, *n* = 24) and no CACTA‐like insertion (purple, *n* = 257).

TE‐GWAS identified one QTL significantly associated with both silique length, with a 3.5‐kb CACTA‐like TE being the peak marker and 1000‐seed weight (**Figure** **5**A).^[^ ^13^ ^]^ Subsequently, TE‐eQTL analysis also identified an eQTL with the 3.5‐kb CACTA‐like TE being significantly associated with the expression level of *BnaA09.CYP78A9*. The colocalization of both QTLs suggest that the CACTA‐like TE is the causal variation for silique length and for the expression level of *BnaA09.CYP78A9* (Figure 5B–D). *CYP78A9* encodes a cytochrome P450 monooxygenase, and it has been found that CYP78A9 may generate signals that activate or promote fruit development, thereby enhancing fruit growth.^[^ ^33^, ^34^, ^35^ ^]^ In rapeseed, previous study has verified that *BnaA09.CYP78A9* affects silique length by promoting cell elongation in silique valves.^[^ ^13^ ^]^ Colocalization analysis showed a CACTA‐like insertion at 3.9‐kb upstream of *BnaA09.CYP78A9* was the causal variant responsible for variations in gene expression of *BnaA09.CYP78A9* and phenotypes (Figure 5D). Accessions with the CACTA‐like TE insertion have significantly higher expression levels of *BnaA09.CYP78A9*, longer siliques and larger 1000‐seed weights compared to those without this TE insertion (Figure 5E–H), suggesting that this CACTA‐like TE insertion can enhance *BnaA09.CYP78A9* expression, leading to increased silique length and 1000‐seed weight, which is consistent with the previous results that CACTA‐like TE can act as an enhancer to stimulate high gene expression and silique elongation.^[^ ^13^ ^]^

The CACTA‐like TE is exclusively distributed in the semi‐winter rapeseed varieties from China (152 out of the 169 accessions with CACTA‐like TE) (Figure 5I), suggesting that this TE insertion may have been introduced during the genetic improvement of semi‐winter rapeseed varieties in China. Based on the registration years of these semi‐winter rapeseed varieties recorded in previous studies, we found that the CACTA‐like insertion mainly appeared in the varieties developed after 1970 (Figure 5J). To further explore its origin, we genotyped the CACTA‐like TE in the 199 *B. rapa* accessions using the resequencing data,^[^ ^36^ ^]^ and found that this TE insertion exists in multiple *B. rapa* subgroups (Figure S10, Supporting Information). These results suggest that the CACTA‐like TE may have been introduced into modern Chinese rapeseed varieties during the process of improvement through distant hybridization between *B. napus* and *B. rapa*, resulting in increased silique length and 1000‐seed weight.

### 2.6 Characterization of the Enhancer Sequence of the CACTA‐Like TE

**Figure 6:**
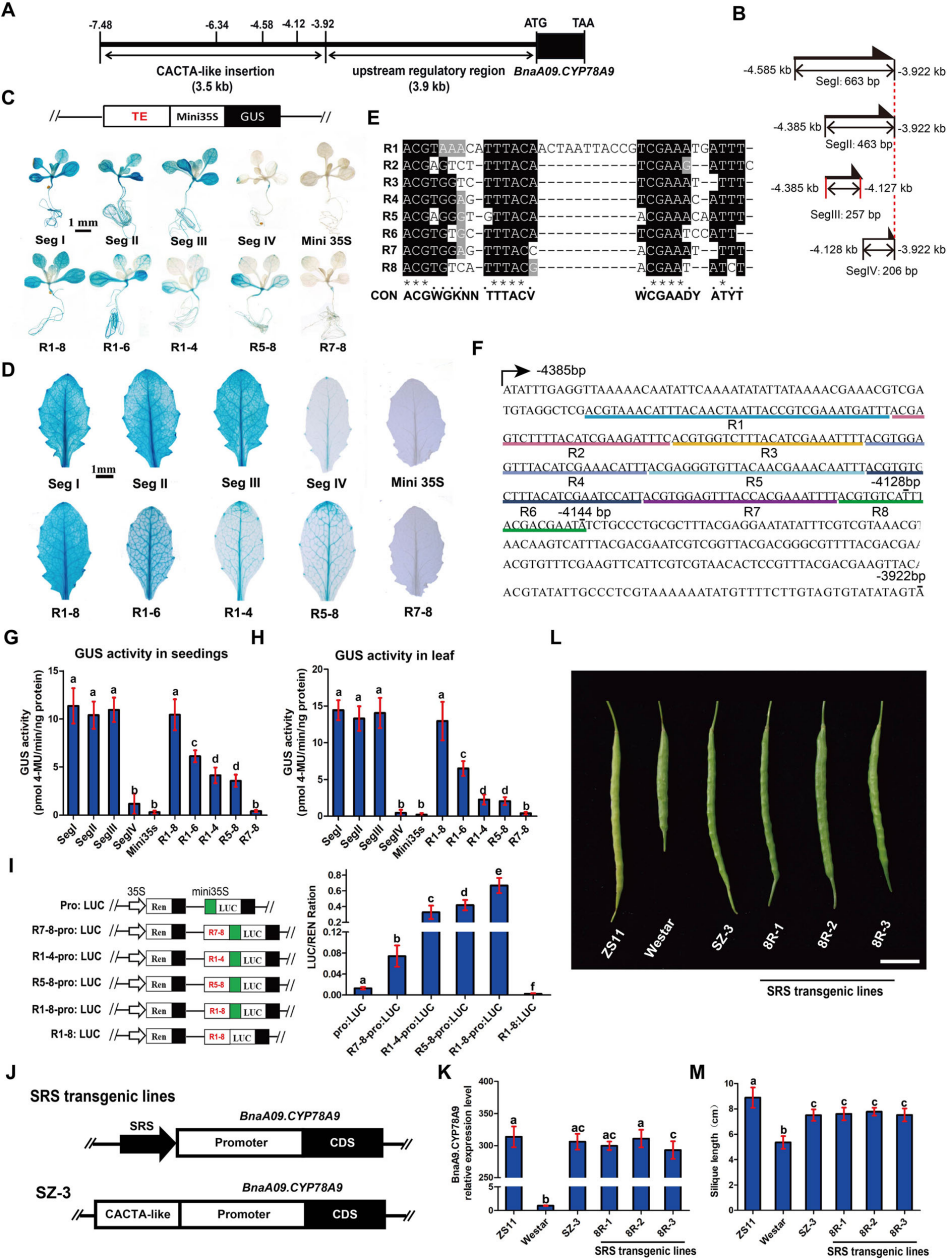
Eight tandem repeats (SRS) within the CACTA‐like element act as an enhancer. A) Schematic diagram of the 3.5‐kb CACTA‐like transposable element inserted into the upstream regulatory area of *BnaA09.CYP78A9*. B) Truncated sequence of CACTA‐like elements in the pCambia3301‐mini35s::GUS vector. C,D) Truncation analysis of the enhancement effect of CACTA‐like elements in 10‐day‐old seedlings (C) or 25‐day‐old leaves (D) of Arabidopsis. The Minimal 35S (mini35S) promoter only was used as a negative control. Bar = 1 mm in (C) and (D). E) DNA sequence alignments of the eight tandem repeats within the CACTA‐like element. Black and gray backgrounds indicate conserved nucleotides, and identical nucleotides are marked with asterisks. F) Nucleotide sequences of Seq ii (463 bp) within the CACTA‐like element. SRS is indicated by underlines with different colors. G,H) GUS activity in transgenic *Arabidopsis* harboring constructs of the minimal 35S promoter fused to different CACTA‐like TE sequences. Quantitative analysis was conducted in 10‐day‐old seedlings (G) and 25‐day‐old leaves (H). I) The LUC/REN ratio shows the effects of SRS enhancer on gene expression. SRS (R1‐8), truncated SRS plus mini35S promoter, mini35S alone, or SRS (R1‐8) alone were fused with LUC. The green box indicates the mini35S promoter. Error bars indicate SD. Bars with different letters represent significant differences at *p‐*value <0.05, *t*‐test. J) Schematic diagram of SRS and the *BnaA09.CYP78A9* coding sequence and its promoter used in the binary transformation vector. K) The qPCR analysis of *BnaA09.CYP78A9* expression levels in transgenic rapeseed. Transcript levels from the transgene‐negative plants (negative) were set to 1. 8S‐1, 8S‐2, and 8S‐3 were three SRS transgene‐positive T1 lines. SZ‐3 harboring the 3.5‐kb CACTA‐like TE, *BnaA09.CYP78A9*, and its promoter was used in the previous complementation test. Error bars indicate SD from three independent experiments. ^**^ *p* value <0.01, *t*‐test. L) Silique phenotype of ZS11, negative lines, and transgenic lines at 30 days post‐anthesis. M) Silique length of ZS11, negative lines, and transgenic lines at 30 days post‐anthesis. Bars with different letters represent significant differences at *p* value <0.05, *t*‐ test.

Previous sequence truncation analysis has delimited that the primary enhancer domain to a fragment of 664 bp ranging from −4,585 to −3,922 bp upstream of the translation start site (TSS) of *BnaA09.CYP78A9* (**Figure** **6**A).^[^ ^13^ ^]^ To further characterize the enhancer core element in this TE, we further dissected the 664 bp fragment into four segments, Seg I (−4585 to −3922 bp), Seg II (−4385 to −3922 bp), Seg III (−4385 to −4127 bp), and Seg IV (−4128 to −3922 bp) (Figure 6B). These segments were fused to the CaMV 35S mini promoter and transformed into Arabidopsis plants. Transgenic plants harboring Seg I, Seg II, and Seg III exhibited similar intensities of β‐glucuronidase (GUS) signals, while plants harboring Seg IV exhibited much weaker GUS signal (Figure 6C,D), indicating that the major part of the enhancer domain is in Seg III, and the minor part is in Seg IV. And eight imperfect tandem repeats (R1‐8) with a 25‐bp consensus sequence were identified in these two regions (Figure 6E). To test the sufficiency of these repeats for enhancer activity, we fused only the eight repeats (R1‐8) with the CaMV 35S mini promoter and transformed into Arabidopsis plants (Figure 6F). Transgenic plants harboring the tandem repeats displayed almost the same intensities and patterns of the GUS signals as the transgenic plants harboring Seg I, II, or III (Figure 6C,D), suggesting that the eight tandem repeats are the core enhancer element within the CACTA‐like TE. We also compared the activities of two, four, six, and eight tandem repeats in the enhancer core element and found that the intensities of GUS signals in transgenic plants harboring different number of repeats showed significant differences (Figure 6C,D). Eight repeats have the highest intensities of GUS signals, followed by six repeats and four repeats. Two repeats have the weakest intensities of GUS signals. In addition, the former four repeats (R1‐4) displayed almost the same intensities and expression patterns of GUS signals with the latter four repeats (R5‐8) (Figure 6C,D). Furthermore, the quantitative GUS activity results were consistent with the GUS staining data presented above (Figure 6G,H). These results suggest that the enhancer activity is proportional to the number of repeats in the CACTA‐like TE and the enhancer activity of different repeat units do not have difference. We further validate the effects of the number of repeats on enhancer activity using transient expression assay with dual LUC genes (Figure 6I). Two (R7‐8), four (R1‐4 or R5‐8), or eight (R1‐8) repeats were fused with the CaMV 35S mini promoter, respectively, to drive the expression of the firefly LUC reporter gene in Arabidopsis protoplasts with the Renlila luciferase gene as an internal control (Figure 6I). The activities of luciferase in transgenic protoplasts harboring different number of repeats also showed significant differences (*p*‐value <0.05, *t*‐test, Figure 6J). Eight repeats have the highest luciferase activity, followed by four repeats, with two repeats having the lowest luciferase activity, which is consistent with the intensities of GUS signals. The eight repeats (R1‐8) only (without the CaMV 35S mini promoter) could not activate the expression of the firefly luciferase gene (Figure 6J), suggesting that the tandem repeats only act as an enhancer to regulate target gene expression. In view that the CACTA‐like TE regulates silique length, we named the eight tandem repeats as Silique Regulation Sequence (SRS).

To validate if the eight repeats confer the same enhancer activity as the whole CACTA‐like TE, we fused the eight repeats (SRS) with the 3.9‐kb native promoter to drive *BnaA09.CYP78A9* in transgenic Westar (a variety with short siliques) plants (Figure 6G), and compared the expression level of *BnaA09.CYP78A9* with the transgenic Westar plants harboring the whole CACTA‐like TE, the native 3.9‐kb promoter and *BnaA09.CYP78A9* created previously.^[^ ^37^ ^]^ The expression levels of *BnaA09.CYP78A9* in three SRS transgenic plants (8S‐1, 8S‐2, and 8S‐3 lines) are similar to that in the whole CACTA transgenic plants and wild‐type ZS11 plants, but significantly higher than that in Westar (Figure 6H). Silique length of three SRS transgenic lines is almost similar to that of ZS11 with long siliques, but significantly longer than that of Westar with short siliques (Figure 6H–J). These results confirmed that the eight repeats are the core element of the enhancer in the CACTA‐like TE and enhancer activity is proportional to the number of repeats.

### 2.7 Regulatory Mechanism for the CACTA‐Like Insertion Enhancing Gene Expression

**Figure 7:**
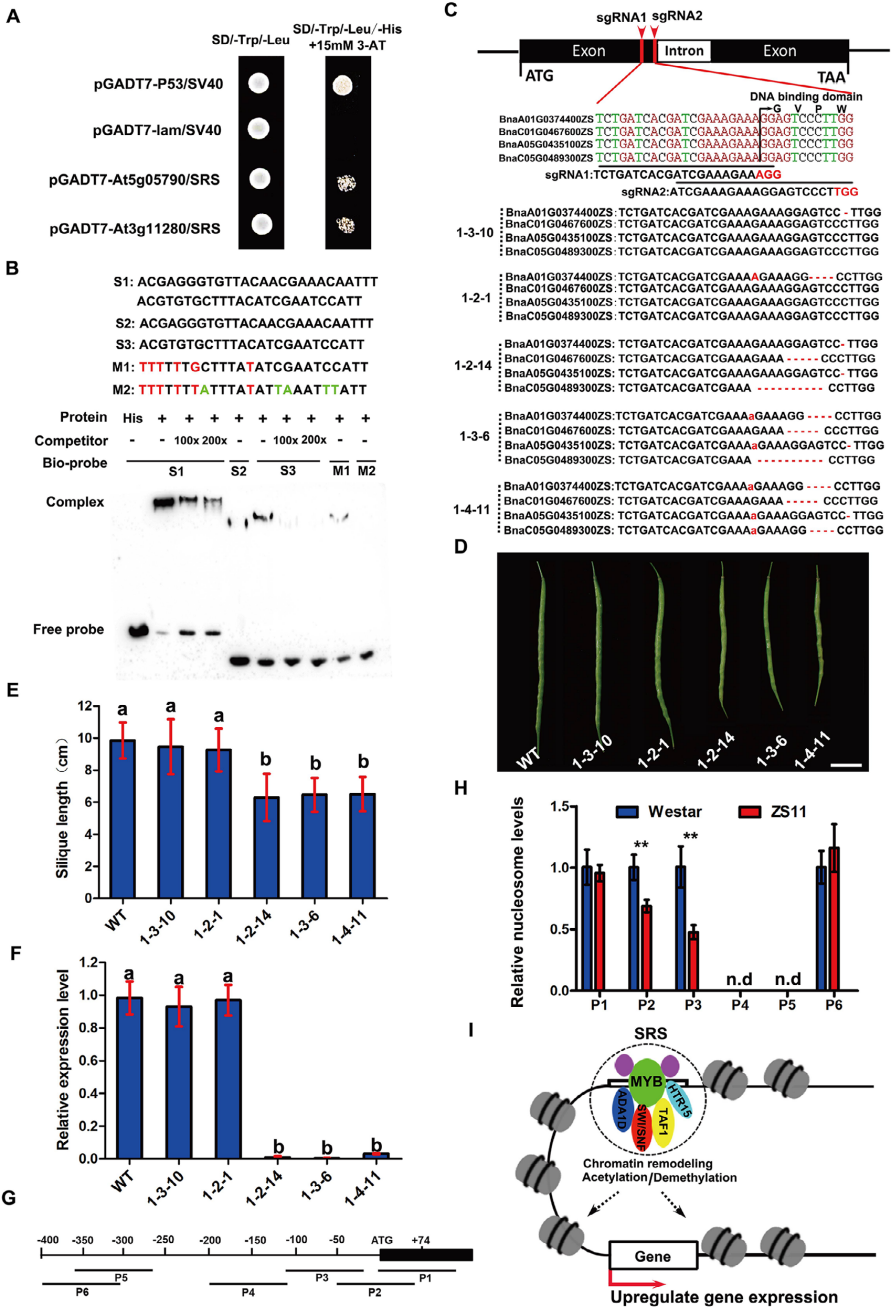
MYB TF binds to SRS, increasing *BnaA09.CYP78A9* gene expression. A) SRS interacting proteins identified by yeast one‐hybrid assay. Transformants were grown on the SD/‐Trp/‐Leu medium and SD/‐Trp/‐Leu/‐His + 15 × 10^−3^ m 3‐AT medium. Transformants of SV40 and pGADT7‐lam were used as negative controls, while transformants of SV40 and pGADT7‐p53 were used as positive controls. B) Gel shift assays revealed that the BnaA01G0374400ZS protein binds to oligonucleotides of S1, S2, and S3, resulting in 100‐ or 200‐fold reductions in excess corresponding cold probes. M1 and M2 were mutations of S3. C) Gene structure of BnaA01G0374400ZS and its homologs, sgRNA sequences, and mutation positions of five independent lines. Black boxes indicate exons, white boxes indicate introns, and black lines indicate untranslated regions. Arrows point to sgRNA target sites. D) Silique phenotypes of WT and mutant lines at 30 days post‐anthesis. Bar = 1 cm. E,F) Silique length (E) and relative expression levels (F) of *BnaA09.CYP78A9* between WT and mutant lines at 30 days post‐anthesis. Error bars indicate standard deviation (SD; *n* > 20). Bars with different letters represent significant differences at *P* value <0.05, *t*‐ test. G) Regions assayed for nucleosome occupancy in regulation and coding areas of *BnaA09.CYP78A9* using MNase‐qPCR. H) MNase‐qPCR shows relative nucleosome levels at the indicated regions in siliques of ZS11 and Westar. Error bars indicate SD from three independent experiments. ^**^ *p* value <0.01, *t*‐test. I) A working model of SRS‐activated transcription in rapeseed.

Enhancers are distal *cis*‐regulatory DNA elements that recruit TFs to increase gene expression.^[^ ^38^ ^]^ To further ascertain the regulatory mechanism of the CACTA TE activating transcription, a high‐throughput screening based on the yeast one‐hybrid method was first performed to identify binding TFs.^[^ ^39^ ^]^ The 211 bp SRS was used as bait to screen the *Arabidopsis* TF library containing 1589 TFs.^[^ ^39^ ^]^ Two MYB TFs (AT5G05790 and AT3G11280) with high similarity were identified to interact with the 211‐bp SRS (**Figures** **7**A, S11A, Supporting Information). Then, four putative orthologs of AT5G05790 and AT3G11280 in the *B. napus* genome of ZS11 were identified, including BnaA01G0374400ZS, BnaA05G0435100ZS, BnaC01G0467600ZS, and BnaC05G0489300ZS. Multiple sequence alignment of AT3G11280 and its orthologs in ZS11 genome demonstrated that all orthologs contain two highly conserved MYB DNA binding domains (Figure S11A,B, Supporting Information), suggesting that they play a role in interacting with the SRS.

To ascertain whether MYB TF directly binds to elements of the repeats within the CACTA‐like TE, we performed electrophoretic mobility shift assays (EMSAs). As the rapeseed ortholog, BnaA01G0374400ZS with the lowest similarity to AT3G11280 was selected to produce His‐tagged recombinant protein, and the R5 and R6 repeats in SRS were randomly selected to produce biotin labeled probes. The His‐tagged recombinant BnaA01G0374400ZS protein was found to bind not only to S1 probe (R5‐6), but also S2 probe (R5) and S3 probe (R6). However, the visible shifts were significantly reduced in S2 probe and S3 probe with only one repeat, compared to S1 probe two repeats (Figure 7B), indicating that the enhancing activity on gene expression is positively correlated with the number of repeats. Additionally, increasing molar excesses of unlabeled competitor probes also reduced or abolished binding efficiency (Figure 7B). Because MYB TFs are known to bind AACC *cis*‐elements,^[^ ^40^ ^]^ we mutated the all of the four AC nucleotides to TT or AT nucleotides in the S3 probe (M1) and found that the binding shifts were significantly reduced (Figure 7B). Mutating all of the C nucleotides in the M1 probe (M2) completely abolished the binding to MYB (Figure 7B). These findings suggest that the AC or C nucleotides in SRS are important for binding to the MYB TFs.

To confirm if SRS activates gene expression by binding to the MYB TFs in rapeseed, we generated mutants of the four MYBs (BnaA01G0374400ZS, BnaA05G0435100ZS, BnaC01G0467600ZS, and BnaC05G0489300ZS) in rapeseed by CRISPR/Cas9 (Figure 7C). Two sgRNAs (sgRNA1 and sgRNA2) simultaneously targeted to the MYB DNA binding domain within the first exon were selected (Figure 7C). Constructs were produced and transformed into ZS11 plants and transgenic plants were identified by DNA sequencing. Five T2 or T3 mutant lines were obtained, including two single mutants (lines 1‐3‐10 and 1‐2‐1), and three quadruple mutants (lines 1‐2‐14, 1‐3‐6, and 1‐4‐11; Figure 7C), were used for further studies. The two single mutants exhibited similar silique lengths to wild type ZS11 plants (Figure 7D,E). The three quadruple mutants had significantly shorter siliques than ZS11 (*p*‐value <0.05, *t*‐test, Figure 7D,E). We examined the expression levels of *BnaA09.CYP78A9* in siliques of these mutants. The expression levels of *BnaA09.CYP78A9* in the two single mutants were similar to that in ZS11, but significantly decreased in the quadruple mutants (*p*‐value <0.01, *t*‐test, Figure 7F), suggesting that the MYBs act redundantly in regulating silique elongation in rapeseed. Taken together, these results further confirm that the four MYBs act as transcriptional activators which bind to SRS in the CACTA‐like TE to enhance the expression of *BnaA09.CYP78A9*.

To further explore the mechanism by which SRS activates transcription, we used IP‐MS to identify BnaA01G0374400ZS‐interacting proteins in ZS11 and transgenic line S1, and further combined a split LUC system in transiently transfected *Nicotiana benthamiana* leaves to validate these interactions. Totally, eight interactions including BnaA01G0374400ZS with ADA1D, TAF1, HTR11, HTR15, ATHB‐53, HSFA1, HTA13, and CYCT1;5 were identified. Among them, the fluorescence signals showed a strong interaction of BnaA01G0374400ZS with ADA1D, TAF1, HTA13, and CYCT1;5, as well as a weak interaction with HTR11, HTR15, ATHB‐53, and HSFA1 (Figure S12, Supporting Information). Considering that DNA is wrapped around histone octamers to form nucleosome that compact the genome and sterically hinder TF binding to nucleosomal DNA,^[^ ^41^ ^]^ we further explore whether SRS activates transcription by providing TF access to nucleosomal DNA. To capture specific nucleosome positioning and occupancy levels around *BnaA09.CYP78A9*, we performed micrococcal nuclease digestion coupled with quantitative real‐time PCR (MNase‐qPCR) at six regions (P1‐P6) which were located from −402 to +79 bp at 5′ upstream of *BnaA09.CYP78A9* in siliques at 20 days post‐anthesis from ZS11 and Westar (Figure 7G). The significant reduced nucleosome occupancy at P2 (−49 to +48 bp) and P3 (−114 to −29 bp) were observed, respectively (Figure 7H). Both P1 (−6 to +79 bp) and P6 (−402 to −310 bp) exhibited similar occupancy in both ZS11 and Westar (Figure 7H). Both P4 (−200 to −106 bp) and P5 (−376 to −283 bp) were unoccupied in both ZS11 and Westar (Figure 7H). Taken together, these results suggest a simple model (Figure 7I) of SRS‐mediated transcriptional activation in which the SRS within the CACTA‐like element acts as an enhancer and activates transcription by the eviction of the TSS nucleosome that is determined by recruitment of MYB TFs and cofactors, such as remodeler SWI/SNF, ADA1D, TAF1, and HTR3‐15.

## 3 Discussion

Rapeseed is one of the main sources of vegetable oil for human production. TEs occupy more than 50% of the whole rapeseed genome. The rapeseed genome contains A and C subgenomes, in which the formation time and number of TEs vary.^[^ ^12^ ^]^ In this study, we identified TEs in 14 rapeseed accessions, and compared TE content, genome distribution, and insertion time in these genomes. TE insertion sites with different classes in the rapeseed genome show different genomic distribution and insertion time, and the genes with TE insertions are subjected to lower purifying selection pressure. Based on a pan‐TE map constructed from 14 rapeseed genomes, analysis of TE insertion polymorphisms at the population level indicates that most TEs in the rapeseed genome are conserved, and those polymorphic TEs are mainly low‐frequency variations. Selective signal analysis and GWAS based on TE genotypes identified a 5556‐bp insertion containing nine TEs in the intron of *BnaA03.FLC* resulting into early flowering in most spring accessions, suggesting that these TEs may play an important role in the ecotype improvement process of spring accessions. These results also demonstrate that TE plays a key role in shaping the adaptive evolution of plants under stress conditions.

In the rapeseed genome, TEs tend to be distributed in noncoding regions and can affect gene expression by altering regulatory sequences and ultimately induce phenotypic variation. Combining with the previous studies in crops, such as rice and maize, TEs can alter gene transcription, activating, or repressing them, by different means in the previous studies.^[^ ^42^, ^43^, ^44^, ^45^ ^]^ Similar to those found in rapeseed genome, TE insertions can recruit repressive histone modifications in rice, such as H3K9me2, and finally repress gene expression.^[^ ^45^ ^]^ Also, insertion of TEs within a promoter can interfere with transcription, and the silencing of TEs inserted close to genes can result in repression of gene expression in maize, such as those found in *BnaC07.MYB28* of rapeseed genome.^[^ ^17^ ^]^ On the other hand, TEs may contain active promoters or enhancers and affect gene expression in maize.^[^ ^44^ ^]^ Similarly, we found the CACTA‐like TE insertion in the upstream of *BnaA09.CYP78A9* contains an active enhancer and promote the expression levels of nearby genes in this study.

In this study, we explored the impact of TE insertions on gene expression levels from two perspectives: the impact on expression levels of different genes with and without TE insertions in one genome, and the impact of the presence or absence of one TE on the expression level of one nearby gene at the population level. On the one hand, as for one rapeseed genome, we found the expression levels of genes with TE insertions were significantly lower than those of genes without TE insertions, which may be owing to the higher methylation levels of these genes with TE insertions. Considering that these genes without TE insertion are subject to strong negative selection, they may be related to important biological functions, so we deduced that their high expression levels may be the result of long‐term genomic evolution. On the other hand, integration analysis with population‐level gene expression levels indicates that TE mainly influences the expression of nearby genes by *cis*‐regulatory and furthermore, TEs tend to enhance the expression of nearby genes. These results can provide data support for understanding the regulation of TEs on gene expression in plants.

TE is one important source of genomic variation in plant genomes and plays an important role in the genetic improvement of rapeseed. In this study, we take the yield‐related traits of rapeseed as an example to further analyze the process of how TEs affect the expression of key genes and ultimately induce excellent phenotypes. Based on the association analysis of population multi‐omics datasets, one CACTA‐like insertion at upstream of *BnaA09.CYP78A9* promote transcription and finally increase silique length and 1000‐seed weight. Subsequently, transgenic experiments revealed that the core enhancer element is a 463‐bp SRS regulatory element sequence in this TE. SRS element in one CACTA‐like insertion as a typical example that a TE‐derived CRE activates transcription, further experiments revealed that it activates transcription by recruiting TFs and cofactors, which provides access to nucleosome DNA, ultimately regulating silique length and 1000‐seed weight. These results suggest TEs play key roles in genetic improvement process of crop traits, providing support for the development and improvement of molecular markers using TEs in the future.

In summary, this study demonstrates that TEs play an important role in regulating gene transcription and driving local adaptation, speciation, domestication, and breeding of rapeseed through the identification and polymorphism analysis of TEs based on the pan‐genomic level. These results demonstrate that the long‐term effects of TEs on genomes is likely underestimated. TEs not only drive genome evolution, participate in transcriptional regulation, and phenotype formation, but also could be used in the future to assist in the development of molecular markers to improve traits owing to their broad polymorphism. However, considering the limited representativeness of the 14 rapeseed genomes used in this study, more genomic resources are needed to capture more comprehensive TE polymorphism. With the development of genome sequencing technology, we will construct a more comprehensive rapeseed pan‐genome and integrate more omics data to further analyze the roles of TEs in rapeseed genome.

## 4 Experimental Section

### Plant Materials and Growth Conditions

All transgenic Arabidopsis plants are in Columbia‐0 (Col‐0) background. Seedlings were grown in a plant growth chamber at 22 °C under long‐day conditions (16 h light/8 h dark). Rapeseed varieties ZS11 and S1 have long‐siliques and large seeds, and variety Westar has regular siliques and regular seeds. All varieties were grown at the experimental farm in Huazhong Agricultural University in autumn. Transgenic rapeseed plants were grown at an isolated experimental farm exclusive for genetically modified crops at Huazhong Agricultural University.

### Data Collection

For the genome dataset, 14 published *Brassica* *napus* genome assemblies and gene annotations were collected (Table S1, Supporting Information). As for the transcriptome dataset, gene expression levels from 2791 RNA‐seq libraries were collected from BnIR database (including 91 libraries from BnTIR).^[^ ^46^ ^]^ As for the phenotypic data involving flowering time, silique length and 1000‐seed weight, they were downloaded from BnVIR. The epigenetic signal datasets were downloaded from BnIR. Resequencing datasets of 2311 accessions were collected from BnVIR database.^[^ ^47^ ^]^

### Construction of the Protein‐Coding Gene‐Based Pan‐Genome

OrthoFinder (v.2.5.4) with default parameters was used to identify orthologous gene clusters among 14 *B. napus* accessions.^[^ ^14^ ^]^ Gene clusters were classified into four categories: those containing members from all 14 *B. napus* accessions were defined as core gene clusters, those containing members from 12 to 13 accessions were defined as softcore gene clusters, those with members from 2 to 11 *B. napus* accessions were defined as dispensable gene clusters, and those present in only one accession were defined as private gene clusters. The simulation of pan‐genome size, measured by the number of protein‐coding genes, was carried out using PanGP (v1.0.1) with the totally random algorithm.^[^ ^48^ ^]^ For each genome, 500 combinations and 30 repeated sampling times were used.

### Construction of Rapeseed Graph Genome

The 14 rapeseed genomes, the Minigraph‐Cactus (MC) Pangenome Pipeline was used to construct the rapeseed graph genome.^[^ ^49^ ^]^ First, using ZS11 as the backbone of the map genome, Cactus (v2.6.8) was used to perform base‐level alignment of homologous relationships between assemblies,^[^ ^50^ ^]^ and extended the pan‐genome constructed by Minigraph (v0.19).^[^ ^51^ ^]^ Then, vg was used to construct a graphical pan‐genome and generate a VCF file with variation bubbles for the graph genome.^[^ ^52^ ^]^ To obtain different types of variations, the annotate_vcf.py script (https://bitbucket.org/jana_ebler/hprc‐experiments/src/master/genotyping‐experiments/workflow/scripts) was used to decompose and annotate variations in bubbles. As a result of this decomposition, paired VCFs were obtained that provide complementary views before and after decomposition. Variant sites were then classified into SNPs, small InDels, DEL; INS; and MNPs. Specifically, deletion‐type SVs were defined as alleles with length (REF) ≥ 50 bp and length (ALT) = 1 bp; insertion‐type SVs as length (REF) = 1 bp and length (ALT) ≥ 50 bp; and all remaining alleles with length ≥ 50 bp were assigned to complex‐type SVs. Unless stated otherwise, downstream genotyping analyses retained SVs (DEL, INS, and MNP) with allele length ≥ 50 bp.

### Pangenome Size and Growth

To assess the stability and representativeness of 14‐assembly rapeseed pangenome, this work quantified how the graph expands as additional genomes are added. Panacus (v0.4.1, https://github.com/marschall‐lab/panacus) was used to compute ordered growth curves for graph nodes, edges, and total sequence length from the GFA generated by the MC graph.^[^ ^53^ ^]^ The publication‐ready plots with the Panacus‐visualize tool were then produced.

### Identification of Transposable Elements in *B. napus* Genomes

To identify TEs in the 14 rapeseed genomes, Extensive De‐Novo TE Annotator (EDTA) (v2.2.1) was used to generate a non‐redundant de novo TE library for each genome with the parameters ‘–species others –sensitive 1 –step all –anno 1’.^[^ ^54^ ^]^ The transposon library was further processed using the pan‐EDTA (v2.2.1) tool to generate the panTElib library. Then, the panTElib library was used to perform homologous annotation of TEs and process the nested TEs of 14 genomes with the default parameters of by RepeatMasker (v 4.1.2‐p1).^[^ ^55^ ^]^ Then, homologous annotation results were split and TE fragments of more than 80 bp were retained as the final TE annotation results. To estimate the continuity of each assembled repetitive element, the LTR assembly index (LAI) was calculated for the complete LTR dataset using LTR_retriever (v2.8).^[^ ^56^, ^57^ ^]^ LAI was used for determining which genome corresponds to the location information of the alt sequence in the subsequent SV.

### TE Insertion Time Estimation

Insertion Time Analysis of Full‐Length LTR Retrotransposons: In this study, full‐length LTR elements were identified and annotated by the EDTA (v2.2.1) pipeline. The LTR_retriever module was integrated in this pipeline for high‐confidence detection of structurally intact LTR elements. This pipeline integrates the LTR_retriever module to enable high‐precision detection of structurally intact LTR elements. Insertion times of full‐length LTRs were calculated based on sequence divergence between the 5′and 3′ LTR pairs. The average substitution rate (*r*) was set as 1.5 × 10^−8^ substitutions per synonymous site per year.^[^ ^23^ ^]^ Insertion times for each intact LTR element were directly extracted from the output file genome.fa.mod.pass.list for downstream analysis. Full‐length LTR elements were classified into three categories: Copia, Gypsy, and Total. The “Total” category includes all intact LTRs, regardless of classification (Copia, Gypsy, or unknown). All elements were grouped by genome and subgenome (A_n_, C_n_). The distribution of insertion times was summarized and visualized using the R packages ggplot2 and dplyr. Smoothed curves were generated by LOESS fitting to represent the trend of LTR abundance over time.

### Genotyping of Population‐Level SVs

Based on the variant bubbles generated by MC from the 14 rapeseed genomes, multiple steps were performed for filtering these variations. First, variants with the missing ratio of more than 0.5 were filtering out. Next, variants with an LV value greater than 0 and the maximum reference sequence length exceeded 100 kb were excluded using vcfbub (https://github.com/pangenome/vcfbub, v0.0.1) with the parameters “‐l 0 ‐r 100 000”. Additionally, variants containing unknown bases (N) in the reference or alternate sequences were filtered out. Then, annotate_vcf.py script (https://bitbucket.org/jana_ebler/hprc‐experiments/src/master/genotyping‐experiments/workflow/scripts/) was used to decompose these variants in bubbles, and structural variants of longer than 50 bp were retained and used for building genotype mapping index file using PanGenie (v3.0.0) with the parameters “‐l 0 ‐r 100 000”.^[^ ^16^ ^]^ Then, genome sequencing reads from 2311 rapeseed accessions were mapped to the graph genome to obtain SV genotypes of these accessions using PanGenie. Finally, SV genotypes were imputed using Beagle (v4.1, Java‐1.8.0_92) with default parameters.^[^ ^58^ ^]^

### Construction of Pan‐TE Map

Based on the population SV genotypes, genomic positions of each SVs in the non‐reference sequences were obtained using halLiftover (v2.2) based on the hal file generated by MC.^[^ ^59^ ^]^ Then, these genomic regions was validated and corrected using BLAST+ (v2.9.0).^[^ ^60^ ^]^ Next, TEs were genotyping according to SV genotypes. For example, as for one TE in one SV, when the region overlapped with this SV cover more than half of this TE, it was associated with this SV. If one TE was associated with more than one SV, all SV genotypes were then merged as the genotype of this TE.

### Genome‐Wide Association Study

As for 18 collected phenotypic datasets, GWAS was performed based on TE genotypes. First, TEs with a minor allele frequency (MAF) lower than 0.05 were filtered. Then, GWAS was performed using GEMMA (v0.98.1). The population structure was controlled by including the first three principal components as covariates, as well as an IBS kinship matrix derived from all variants (SNPs and InDels) calculated by GEMMA. The cutoff for determining significant associations was set as −log10 (1/*n*), where *n* represents the total number of variations. Finally, GWAS loci were fine mapped using Plink (v1.90b4.4) with the parameters “–clump‐snp‐field SNP –clump‐field p –clump‐kb 100 –clump‐p1 1e‐5 –clump‐r2 0.1”.^[^ ^61^ ^]^ As for each locus, genomic regions were merged according to the positions of all significant TEs in this locus.

As for phenotypic variance explanation (PVE) analysis for 18 phenotypes, SNP, SV, and TE genotypes were pruned respectively using Plink (v1.90b4.4) with the parameters “–indep‐pairwise 100 10 0.8”. Then, PVE values of SNP, SV, and TE genotypes were calculated respectively using GCTA (v1.92.4) with the parameters “–reml”.

### RNA‐seq Analysis

As for two population‐level transcriptome datasets from seeds at 20 and 40 days after flowering, TrimGalore (v0.6.6) was used for adapter trimming and quality control with the parameters “–paired –quality 20 ‐a AGATCGGAAGAGC ‐a2 AGATCGGAAGAGC –length 20”. Then, clean reads were mapped to the ZS11 reference genome using STAR (v2.7.8a) with the parameters “–twopassMode Basic –outSAMtype BAM Unsorted”.^[^ ^62^ ^]^ SAMtools (v1.9) was used to sort the mapped reads.^[^ ^63^ ^]^ To eliminate false‐positive associations during eQTL mapping and correct sequencing biases, WASP (v.0.3.4) was used to correct the mapping result with default parameters.^[^ ^64^ ^]^ Finally, expression levels (transcripts per million, TPM) of genes were quantified using featureCounts (v2.0.0).^[^ ^65^ ^]^

### DNA Methylation Analysis

The WGBS datasets from two accessions (B409 and 2063A) at different tissues and development stages were downloaded from the previous study.^[^ ^25^ ^]^ TrimGalore (v0.6.6) was used for adapter trimming and quality control with the same pipeline as that used in RNA‐seq study. Then, clean data of each accession were mapped to the ZS11 reference genome using Bismark (v.0.13.0) with the parameter settings “‐N 1, ‐L 30”. Next, the average methylation ratio of the three cytosine methylation contexts (‌CG‌, ‌CHG‌, and ‌CHH‌) within each gene was calculated to represent its methylation levels. To visualize methylation profiles by gene‐body TE status, genes were divided into two groups: the genes with TE insertions (TE⁺), defined as genes with at least one TE overlapping the gene body (UTRs/exons/introns between TSS and TES), and genes without TE insertions (TE^−^), defined as genes with no TE overlap within the gene body. For each accession and context (CG, CHG, CHH), metagene profiles were generated over the gene body only, scaled to a common length and partitioned into 60 equal‐width bins. Profiles were then plotted for CG, CHG, and CHH contexts using deepTools (v3.5.6).^[^ ^66^ ^]^

### Data Analysis of Chromatin Immunoprecipitation Followed by Sequencing (ChIP‐seq)

As for the downloaded ChIP‐seq datasets, the clean reads were mapped to the ZS11 genome using bowtie2 (v.2.3.2) with default parameters.^[^ ^67^ ^]^ Next, PCR duplicated reads were removed using Picard (v.2.19, http://broadinstitute.github.io/picard/). Peaks were called using the callpeak module of MACS2 software (v.2.1.2) with the parameters “‐broad ‐f BAM ‐g 1 000 000 000 ‐B ‐p 0.00001 ‐nomodel ‐extsize 147”.^[^ ^68^ ^]^

### eQTL Mapping

For eQTL mapping, genes with TPM values below 0.01 in more than 80% of the samples were first excluded. To correct bases in sequencing depth of different accessions, gene expression levels were normalized using the TMM (trimmed mean of M‐values) method.^[^ ^69^ ^]^ And the top 10 principal components of the expression data were used as covariate for eQTL mapping. On the other hand, TE genotypes were filtered, and TEs with a MAF threshold of more than 0.05 and a heterozygosity threshold of less than 0.5 were retained for eQTL analysis. For the eQTL analysis, *cis*‐eQTLs (within 1 Mb of the gene) were identified using *cis* module in QTLtools (v1.3.1) with the parameters “–nominal 1 –windows 1 000 000 –std‐err”.^[^ ^70^ ^]^ And *trans*‐eQTLs (more than 1 Mb from the gene) were identified using *trans* module in QTLtools with the parameters “–nominal –window 1 000 000”. Similar to GWAS, the cutoff for determining significant associations was set as ‐log_10_ (1/*n*), where n represents the total number of variations, and clumping analysis was performed using Plink (v1.9) to identify lead TEs in TE‐eQTLs.^[^ ^61^ ^]^

### Analysis of TE and Adjacent Gene Expression Levels

To explore the effect on adjacent gene expression levels of TEs, BEDTools (v2.29.0) was first used to identify the nearest gene for each TE.^[^ ^71^ ^]^ Combining the TE genotypes with gene expression levels from seeds at 20 DAF and 40 DAF, average TPM values of accessions with TE insertion and no TE insertion were compared. If the expression level of accessions with TE insertion is at least 1.5 times higher than those with no TE insertion, these transposons are classified as promotive TEs. Conversely, If the expression level of accessions with TE insertion is at least 1.5 times lower than those with no TE insertion, these transposons are classified as suppressive TEs.

### Colocalization Analysis

GWAS loci associated with silique length and thousand seed weight and candidate genes in these loci were used for co‐localization analysis using the “COLOC” R package with default parameters.^[^ ^72^ ^]^ The variants in *cis*‐eQTLs of genes and GWAS QTLs of phenotypes were defined as co‐localized when the posterior probability of a co‐localized signal (PPH_4_) value was higher than 0.5 and they shared at least one significant variation.

### GO and KEGG Enrichment Analysis

The GO and KEGG enrichment analysis in this study was similar to that used in the previous study.^[^ ^15^ ^]^ First, blastp program in BLAST+ (v2.9.0) was used to establish homologous gene pairs of ZS11 reference genome and Arabidopsis genome.^[^ ^60^, ^73^ ^]^ Then, ZS11 genome assembly GO and KEGG annotations were assigned to genes in ZS11 genome according to the GO and KEGG annotation results of the Arabidopsis genome. Next, GO and KEGG libraries were established for ZS11 genome using the “clusterProfiler” R package.^[^ ^74^ ^]^ Finally, based on the GO and KEGG libraries, the “clusterProfiler” R package was used for GO and KEGG enrichment analysis.

### Sequence and Phylogenetic Analyses

The protein sequences of *BnaA01G0374400ZS* and its orthologs were acquired from the NCBI database (http://www.ncbi.nlm.nih.gov) after performing a BLASTP search. Multiple protein sequence alignment was carried out with ClustalX,^[^ ^75^ ^]^ and was refined manually. A phylogenetic tree was constructed using the neighbor‐joining (N–J) method in MEGA (v4.0).^[^ ^76^ ^]^

### Histochemical Analyses of GUS Activity

GUS staining was performed as previously described.^[^ ^77^ ^]^ Briefly, seedlings and leaves were immersed in assay buffer at 37 °C for 12 h followed by washing with 95% ethanol. The GUS activity was examined using a M205A stereomicroscope (Leica, Germany). All experiments were repeated three independent times.

### Plasmid Construction, Arabidopsis, and Rapeseed Transformation

To determine the core enhancer motif, a series of truncations from the CACTA‐like TE of ZS11 were fused with the minimal 35S (mini35S) promoter and the *GUS* reporter gene and cloned into the PCambia3301 vector. All constructs were transformed into wild‐type Arabidopsis (Col) plants using the floral dip method.^[^ ^78^ ^]^ Homozygous lines were selected for analysis of GUS activity.

To determine whether SRS could increase *BnaA09.CYP78A9* expression in Westar, the *BnaA09.CYP78A9* coding sequence, together with the 3‐kb promoter and SRS enhancer fragment (R1‐8) were inserted into the pCAMBIA2301 vector. To generate the mutation construct, two sgRNAs were designed based on the conserved domain of BnaA01G0374400ZS and its orthologs, and the CRISPR‐Cas9 system was created according to the golden gate method.^[^ ^79^ ^]^

Transgenic rapeseed plants harboring each of these plasmids were created by using *Agrobacterium*‐mediated transformation.^[^ ^80^ ^]^ The detected mutations of BnaA01G0374400ZS and its orthologs were confirmed using DNA sequencing. Primers are listed in Table S7 (Supporting Information).

### Yeast One‐Hybrid Assay

The yeast one‐hybrid assay was performed as previously described.^[^ ^39^ ^]^ Briefly, the SRS enhancer fragment (R1‐8) was cloned into the *pHisi*‐2 vector and then transformed into YM4271 yeast as bait to screen a library of 1589 Arabidopsis TFs.^[^ ^39^ ^]^ The TF library strains and the bait clone carrying the SRS fragment were grown overnight in SD/‐Leu and SD/‐His medium, respectively. Later, equal volumes (20 µL well^−1^) of the library strains and the bait clone were mixed and transferred to a new 2‐ml 96‐well plate with YPDA medium. Mating was conducted by shaking at 200 rpm and 30 °C for 24 h. Immediately after shaking, the mating products were diluted with water, plated on selective plates [SD/‐Leu/‐His+15 × 10^−3^ m 3‐aminotrizole (3‐AT)], and incubated for 3 days.

The Y1H assay was performed as previously described.^[^ ^81^ ^]^ Briefly, the SRS enhancer fragment (R1‐8) was cloned into the pHIS‐2 vector as bait. The coding sequences of the TFs AT5G05790 and AT3G11280 were separately cloned into pGADT7 vector as prey. The bait plasmid pHIS‐2‐SRS and pGADT7‐TF were co‐transformed into yeast strain Y187 using the lithium acetate protocol (Clontech). Then, these yeast cells were cultured on transformation plate containing SD/‐Trp/‐Leu and selective plate containing SD/‐Trp/‐Leu/‐His and 15 × 10^−3^ m 3‐AT for 3 days at 30 °C. The SV40 and pGADT7‐p53 vectors were used as positive controls, and the SV40 and pGADT7‐lam vectors were used as negative controls. Primers are listed in Table S7 (Supporting Information).

### Dual LUC Assay

A series of SRS enhancer fragments coupled with the mini35S promoter, or mini35S or SRS (R1‐8) alone, were cloned into the pGreenII 0800‐LUC vector to drive firefly *LUC* expression, with the Renilla LUC reporter gene (*REN*) as an internal control. Primers are listed in Table S8 (Supporting Information). Arabidopsis protoplasts (Col‐0) were isolated as previously described,^[^ ^82^ ^]^ and constructs were transformed using PEG4000.^[^ ^83^ ^]^ The dual LUC array was detected using an E1910 Dual‐LUC Reporter Assay System (Promega, USA). Three biological and technical replicates were performed for each assay.

### Electrophoretic Mobility Shift Assay (EMSA)

To verify the binding of the BnaA01G0374400ZS protein to the SRS enhancer, EMSA was conducted using a GS009 EMSA kit (Beyotime, China), according to the manufacturer's manual (https://www.beyotime.com). His alone and BnaA01G0374400ZS‐His fusion proteins were produced using *Escherichia coli* BL21 (DE3) strains. Briefly, the recombinant BnaA01G0374400ZS protein was incubated with biotin‐labeled oligos, while unlabeled oligos were annealed for competition and nucleotide substitutions were used for mutations. DNA binding reactions were performed at room temperature for 20 min and the protein‐DNA complexes were separated in a 5% native polyacrylamide gel and transferred to nylon membranes. Positive reactions were detected using a chemiluminescent HRP substrate (Beyotime, China).

### Split‐LUC Complementation Assays

To investigate protein–protein interactions between BnaA01G0374400ZS and cofactors, a split LUC complementation assay was performed with pCAMBIA‐nLUC and pCAMBIA‐cLUC vectors in *N. benthamiana* leaves, according to previously described methods.^[^ ^84^ ^]^ The BnaA01G0374400ZS coding sequence was inserted into pCAMBIA‐nLUC and pCAMBIA‐cLUC, respectively. The coding sequences of *TAF1*, *HRT15*, *HSFA1E*, and *HTA13* were cloned into pCAMBIA‐nLUC, and *ADA1D*, *HRT11*, *ATHB‐53*, and *CYCT1*;*5* were separately inserted into pCAMBIA‐cLUC. The constructs were transformed into *Agrobacterium* strain EHA105, and the *Agrobacterium* strains carrying the nLUC and cLUC vectors were co‐infiltrated into *N. benthamiana* leaves with a syringe. After 48 h of incubation, 0.15 mg mL^−1^ D‐luciferin potassium salt was injected into leaves. Images were captured with a cooled Tanon 5200 Chemiluminescent Imaging System (Tanon, China). Three biological replicates were performed for each assay. Primers are listed in Table S7 (Supporting Information).

### IP‐MS Analysis

Coimmunoprecipitation and mass spectrometry were performed as previously described.^[^ ^85^ ^]^ Briefly, siliques at about 20 days post‐anthesis (dpa) were separately collected from ZS11 and S1 seedlings harboring the SRS enhancer inserted into the *BnaA09.CYP78A9* regulatory sequence. Siliques were ground to powder in liquid nitrogen, and protein complexes were extracted using a protein extraction buffer. The extracted protein complexes were immunoprecipitated using a customized MYB‐TF antibody produced by Dia‐An Biotech (China). Subsequently, the bound proteins were eluted and separated by 10% SDS–PAGE. After samples were digested with trypsin, the peptides were detected using liquid chromatography–tandem MS using an EASY‐nLC 1200 coupled to a Q Exactive Plus mass‐spectrometer. The proteins were identified by searching the UniProt database (https://www.uniprot.org) using MaxQuant (V1.6.6) software, with 1% of false discovery rate.^[^ ^86^ ^]^

### MNase‐qPCR

MNase digestion was performed as previously described.^[^ ^87^ ^]^ Briefly, siliques at about 20 dpa from ZS11 and Westar seedlings were fixed with formaldehyde and neutralization glycine. Samples (2.0 g) were ground in liquid nitrogen, and chromatin was isolated using nuclear extract buffer (20 × 10^−3^ m Tris‐HCl pH7.5, 50 × 10^−3^ m EDTA pH 8.0, 5 × 10^−3^ m spermidine, 0.15 × 10^−3^ m spermine, 40% glycerol, and 0.1% mercaptoethanol), followed with nuclear washing buffer (nuclear extract buffer plus 0.5% Triton X‐100). The purified chromatin was resuspended using micrococcol nuclease (MNase) digestion buffer (20 × 10^−3^ m Tris‐HCl, pH7.5, 15 × 10^−3^ m NaCl, 60 × 10^−3^ m KCl, 1 × 10^−3^ m CaCl_2_, and 4 × 10^−3^ m MgCl_2_). Next, 100 µL of the nuclei was digested using 1 µL of (2 × 10^3^ gel U µL^−1^) MNase (M0247S, New England Biolabs, USA) at 37 °C for 10 min. MNase was inactivated by the addition of 0.5 m EDTA (pH 8.0), and reverse cross‐linking was carried out using 5 m NaCl, 20% SDS, and 1 × TE overnight. The reverse cross‐linked reactions were purified using a phenol:chloroform mixture. Nucleosome levels at each site were normalized to naked genomic DNA. The relative nucleosome occupancy levels at the *BnaA09.CYP78A9* promoter were quantified using the 2^−△△Ct^ method. Primers are listed in Table S7 (Supporting Information).

### RNA Extraction and qPCR

Total RNA was extracted from siliques at about 20 dpa using TRIzol (Invitrogen, USA), according to the manufacturer's instructions. qPCR analysis was carried out using a Roche Real‐Time PCR System using TB Green Premix Ex Taq II (Tli RNaseH Plus, Takara). The rapeseed *Epsin N‐terminal homology* (*ENTH*) gene was used as the internal control. Three biological and technical replicates were performed for each assay. Primers are listed in Table S8 (Supporting Information).

## Conflict of Interest

The authors declare no conflict of interest.

## Supporting information



Supporting Information

Supporting Information

## Data Availability

All genome assemblies, annotations and SV information are available at the BnIR database (https://yanglab.hzau.edu.cn/BnIR). Short‐read data of genome resequencing of 2311 accessions were downloaded from the NCBI BioProject, accession nos. SRP067370, SRP125656 and SRP155312, and the GSA BioProject, accession nos. PRJCA002835 and PRJCA000376. RNA‐seq data of 20‐d.a.p. and 40‐d.a.p. seeds were downloaded from the GSA Bioproject, accession no. PRJCA002836. RNA‐seq data of leaves from eight accessions were downloaded from the NCBI BioProject, accession no. PRJNA546246. Whole genome bisulfite sequencing (WGBS‐seq) dataset from two accessions (B409 and 2063A) were downloaded from the NCBI GEO under accession number GSE143287. All scripts and codes used in the present study are available via Zenodo at https://doi.org/10.5281/zenodo.15240426. All datasets of TE annotations from 14 rapeseed genomes in the present study are available via Zenodo at https://doi.org/10.5281/zenodo.16959683.
