# Supplementary material for: A Pan‐TE Map Reveals the Important Role of Transposable Elements in Gene Expression and Phenotypic Diversity in 2,311 Rapeseed Accessions
Source: Adv Sci (Weinh). 2025 Dec 8;13(4):e12036. doi: 10.1002/advs.202512036 (PMC12822431; doi:10.1002/advs.202512036)
Supplement: Supplementary file 1 — Supporting Information [file ADVS-13-e12036-s001.pdf]

A pan-TE map reveals the important role of transposable elements in gene expression and phenotypic diversity in 2,311 rapeseed accessions

Zhiquan Yang, Haiyan Fan, Yifan Chen, Jiawei Li, Minjian Chen, Yingying Tan, Liang Guo, Jintao Li, Kede Liu and Qing-Yong Yang

Zhiquan Yang, Haiyan Fan, Yifan Chen, Jiawei Li, Minjian Chen, Yingying Tan, Liang Guo, Kede Liu, Qing-Yong Yang

National Key Laboratory of Crop Genetic Improvement, Huazhong Agricultural University, Wuhan 430070, China

Zhiquan Yang, Yifan Chen, Jiawei Li, Minjian Chen, Liang Guo, Qing-Yong Yang

Yazhouwan National Laboratory, Sanya 572025, China

Haiyan Fan, Jintao Li

College of Life Sciences, Xinyang Normal University, Xinyang, 464000, China

E-mail: kdliu@mail.hzau.edu.cn (Kede Liu) and yqy@mail.hzau.edu.cn (Qing-Yong Yang)

Zhiquan Yang, Haiyan Fan and Yifan Chen contributed equally to this work.

Funding: This work was financially supported by the National Natural Science Foundation of China (grant nos. 32441059, 32322061, 32501953), National Key Research and Development Program of China (grant nos. 2021YFF1000100), Knowledge Innovation Program of Wuhan-Basic Research (grant no. 2022020801010221), Fundamental Research Funds for the Central Universities (grant no. 2662022YJ016) and the Basic Research Project in 2023 of Yazhouwan National Laboratory (grant no. GL23YCKY01).

This supplementary information file includes:

Supplemental Figures

Supplementary Figure S1: KEGG enrichment analysis of genes in different gene clusters

Supplementary Figure S2: Genomic distribution of different genomic features

Supplementary Figure S3: Enrichment analysis of genomic features in TE insertion region

Supplementary Figure S4: Analysis of TE insertion time

Supplementary Figure S5: Analysis of polymorphic TEs

Supplementary Figure S6: Summary of relationship between TEs and adjacent gene expression levels

Supplementary Figure S7: Comparison for ratios of promoted and suppressed TEs with two orientations

Supplementary Figure S8: Comparison for ratios of promoted and suppressed TEs across seeds at 20 DAF and 40DAF.

Supplementary Figure S9: Manhattan plot from GWAS of 18 phenotypes

Supplementary Figure S10: Frequency distribution of CACTA-like insertion in 199 *B. rapa* accessions

Supplementary Figure S11: Homologs of AT3G11280 and AT5G05790 in the *Brassica napus* genome

Supplementary Figure S12: Split-luciferase complementation assay confirmed BnaA01G0374400ZS interacting proteins

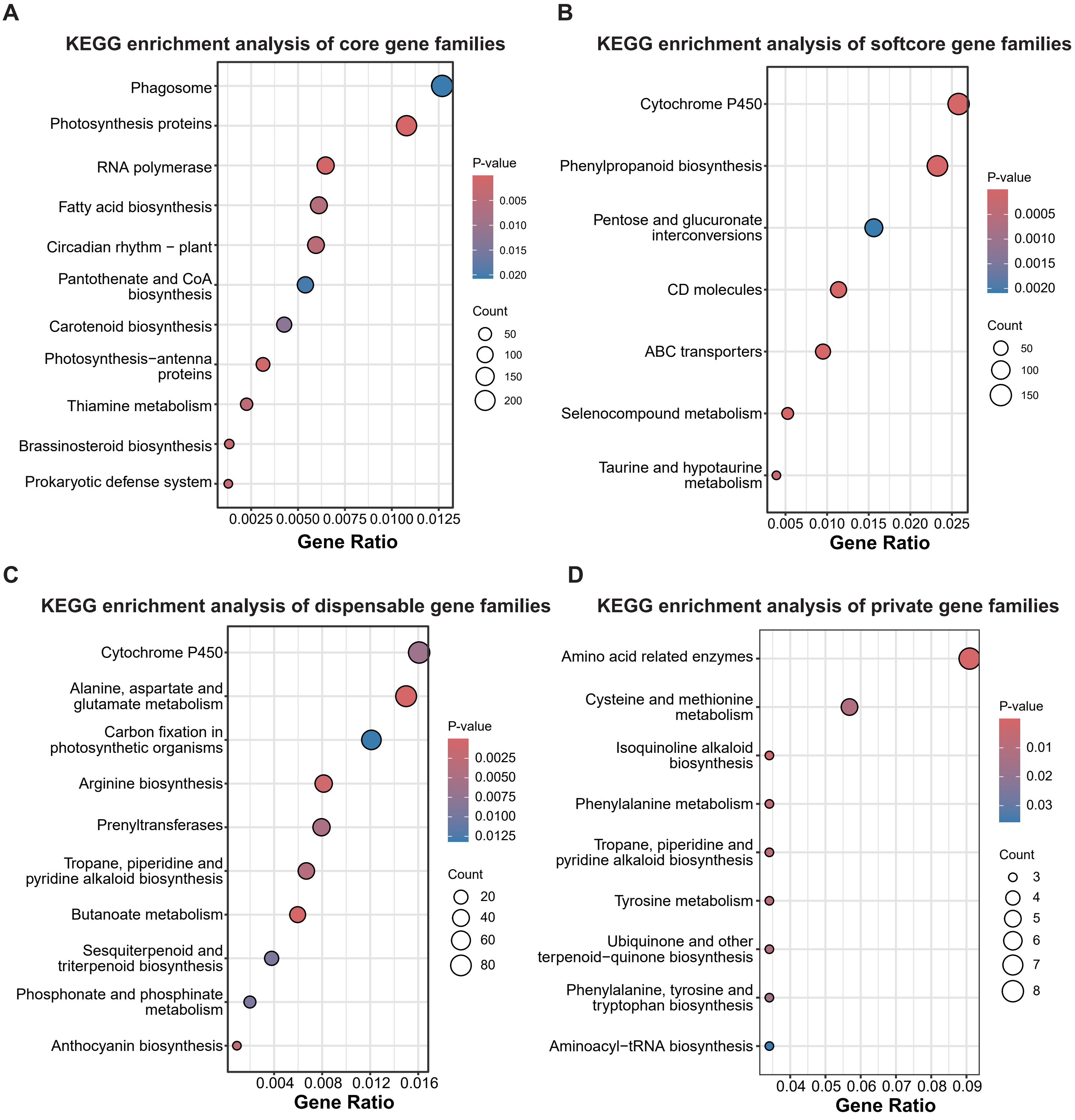

Supplementary Figure S1. KEGG enrichment analysis of genes in different gene clusters. A-D) KEGG enrichment results of core(A), softcore(B), dispensable(C) and private(D) genes. Red to blue circles represent the significance of enrichment analysis and sizes of circles represent gene numbers.

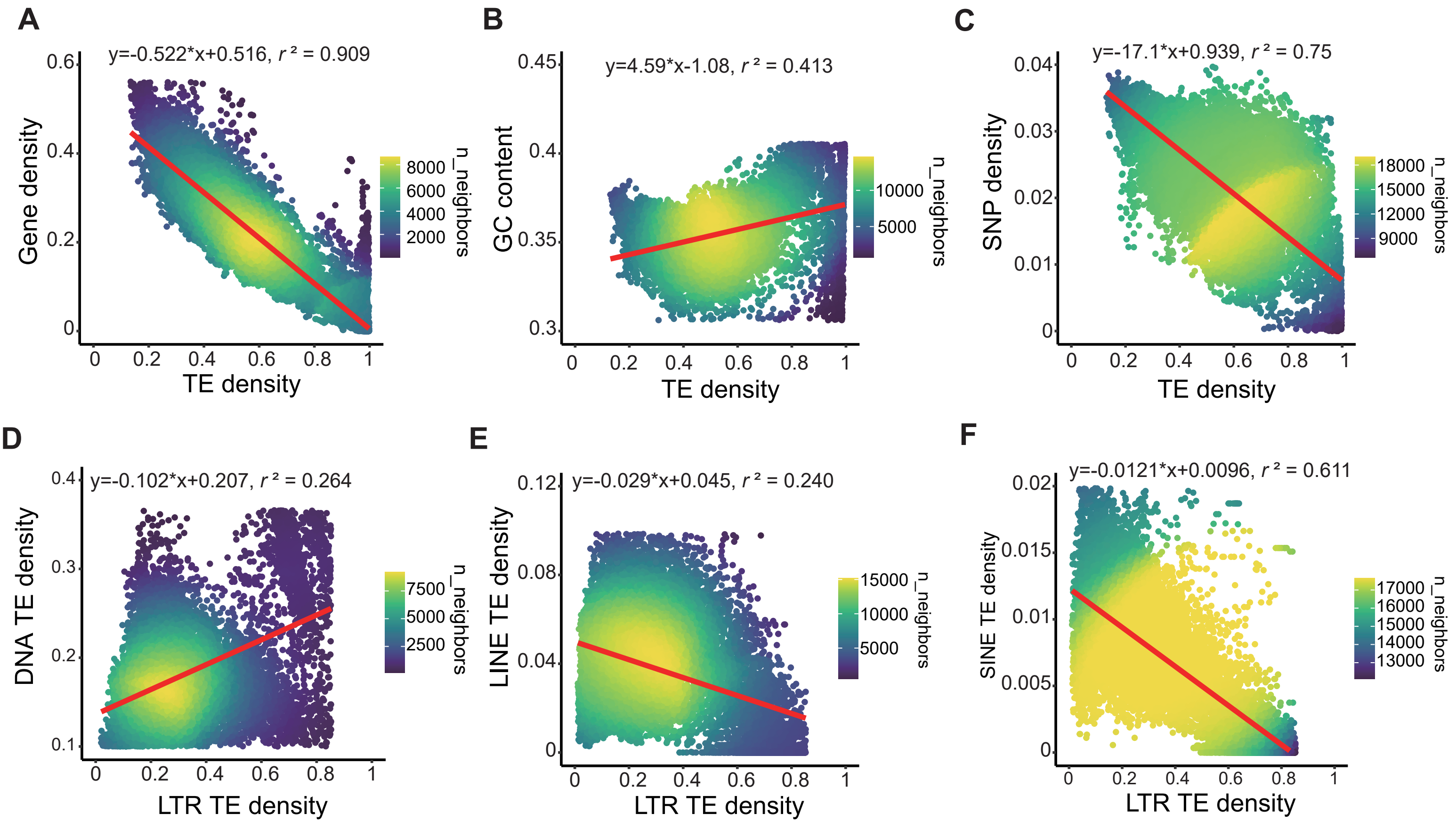

Supplementary Figure S2. Genomic distribution of different genomic features. A-C) Relationship of TE density with gene density (A), GC content (B) and SNP density (C). D-F) Relationship of LTR-TE density with DNA-TE density (D), LINE-TE content (E) and SINE-TE density (F). Blue to yellow dots represent the correlation coefficients of paired features with the window size and step of 500-kb.

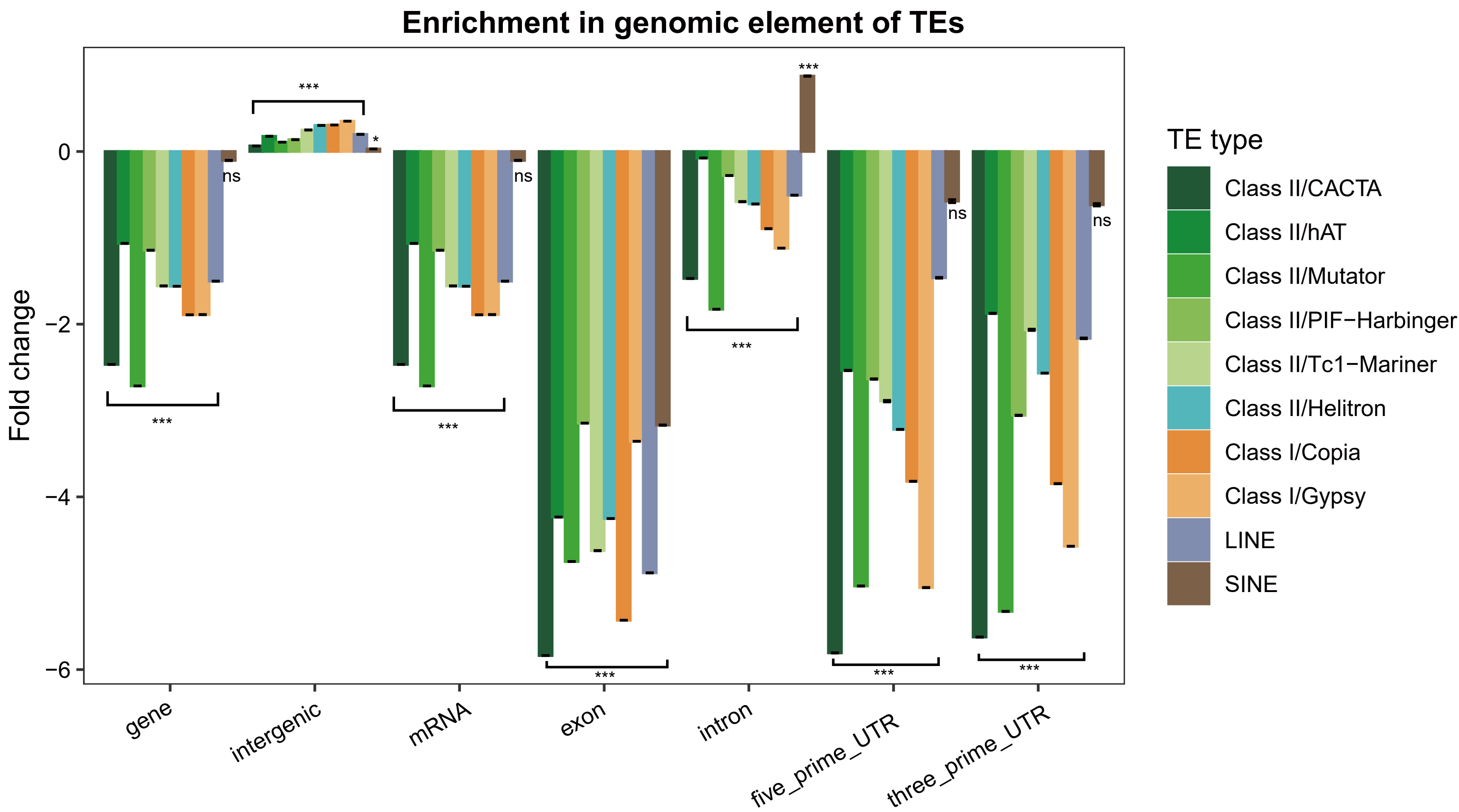

Supplementary Figure S3. Enrichment analysis of genomic features in TE insertion region. Enrichment in genomic elements for TE insertions. Bars with different colors represent TEs of different classes/subclasses. Significance of enrichment analysis assessed via permutation testing. *P*-value was calculated using 1000 permutations.

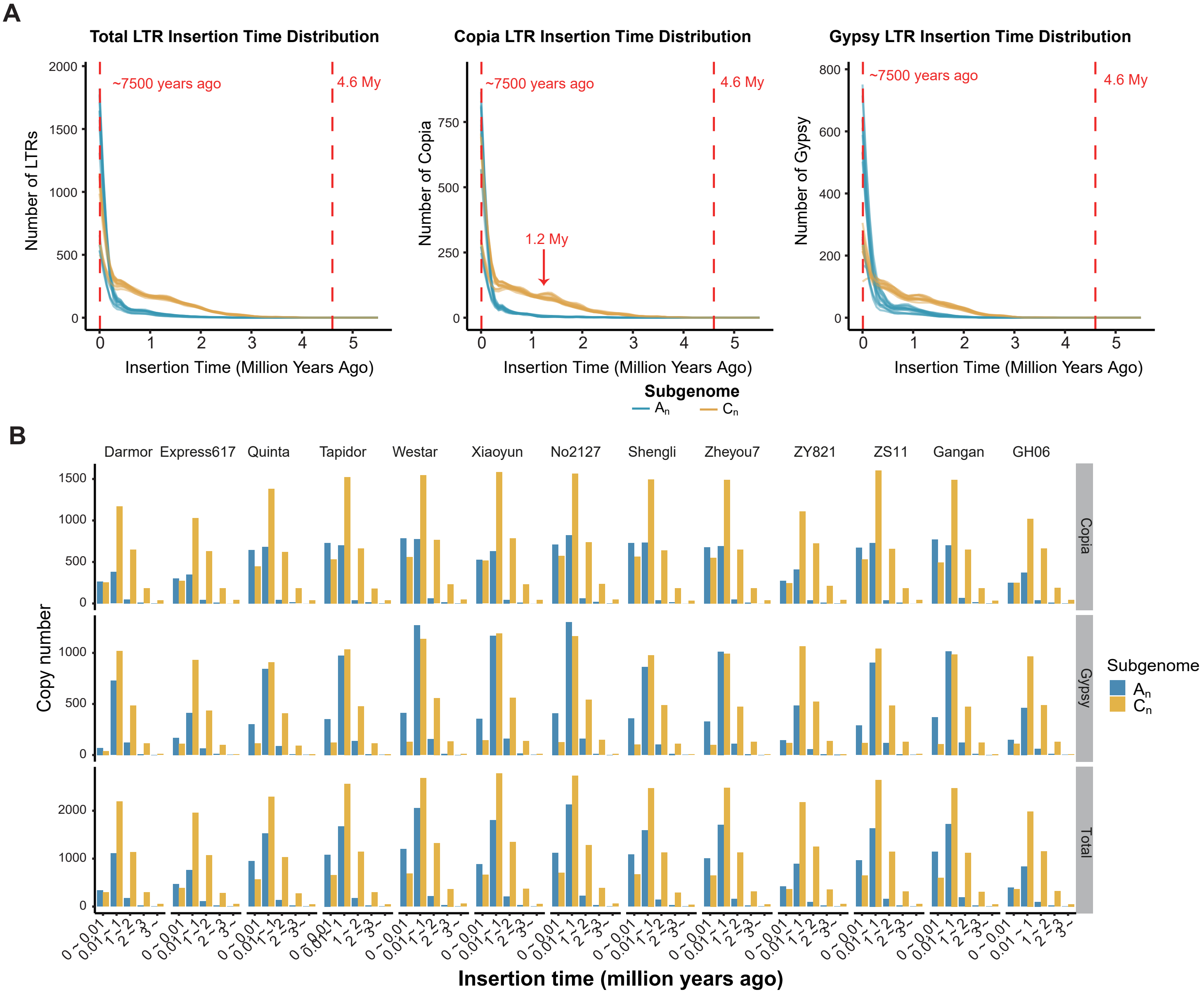

Supplementary Figure S4. Summary for LTR-TE insertion time. A) Distribution of LTR-TE insertion time. Three panels from left to right are insertion time distribution of intact LTR-TEs and two LTR subfamilies (Copia- and Gypsy-TEs), respectively. Blue and yellow lines represent A_n and C_n subgenomes of all rapeseed genomes, respectively. Two dashed red lines represent two re cent genome duplication events including separation of *Brassica rapa* (*B. rapa*) and *Brassica ol eracea* (*B. oleracea*, ~4.6 million years ago) and rapeseed formation (~7,500 years ago). B) S ummary of LTR-TE insertion time. Blue and yellow bars represent A_n and C_n subgenomes of all r apeseed genomes, respectively.

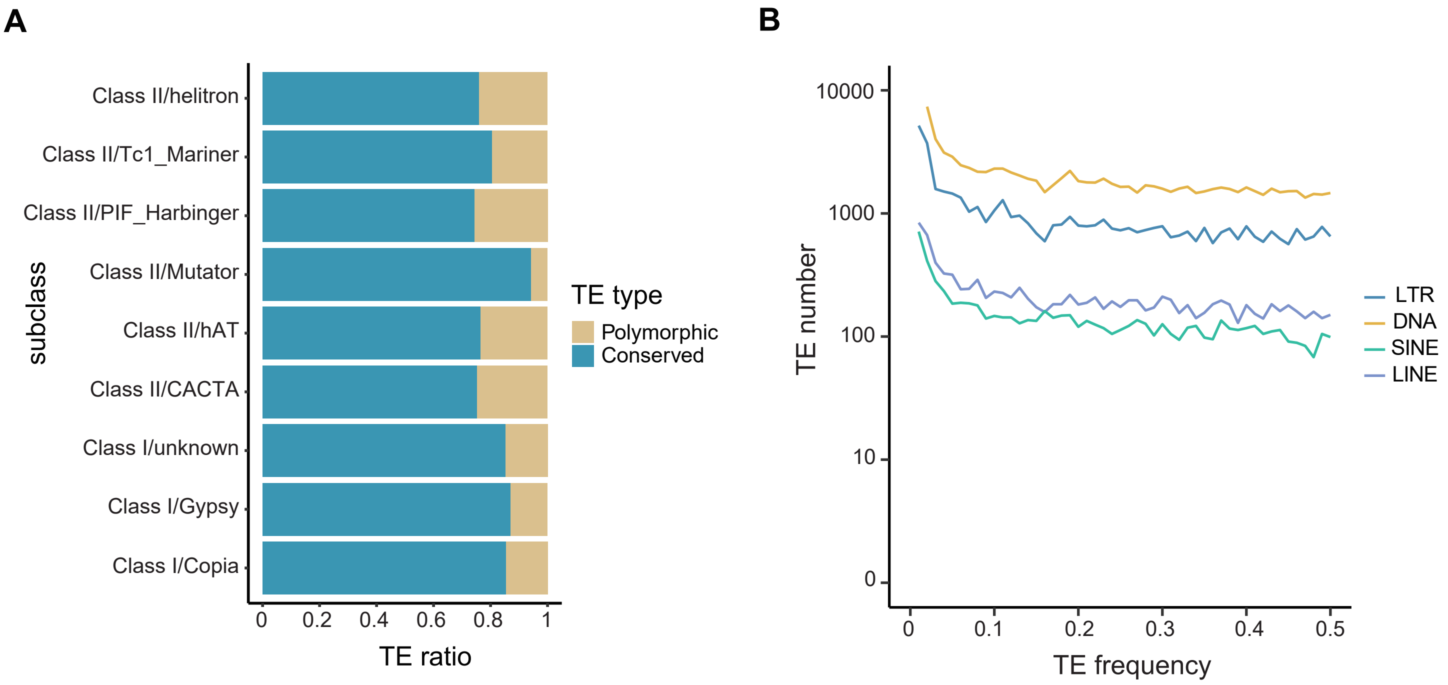

Supplementary Figure S5. Analysis of polymorphic TEs. A) Ratios of polymorphic TEs with different classes and subclasses. Yellow and blue bars represent polymorphic and conserved TEs, respectively. B) Frequency distribution of TEs with different classes. Lines with different colors represent LTR (blue), DNA (orange), SINE (green) and LINE (purple) TEs, respectively.

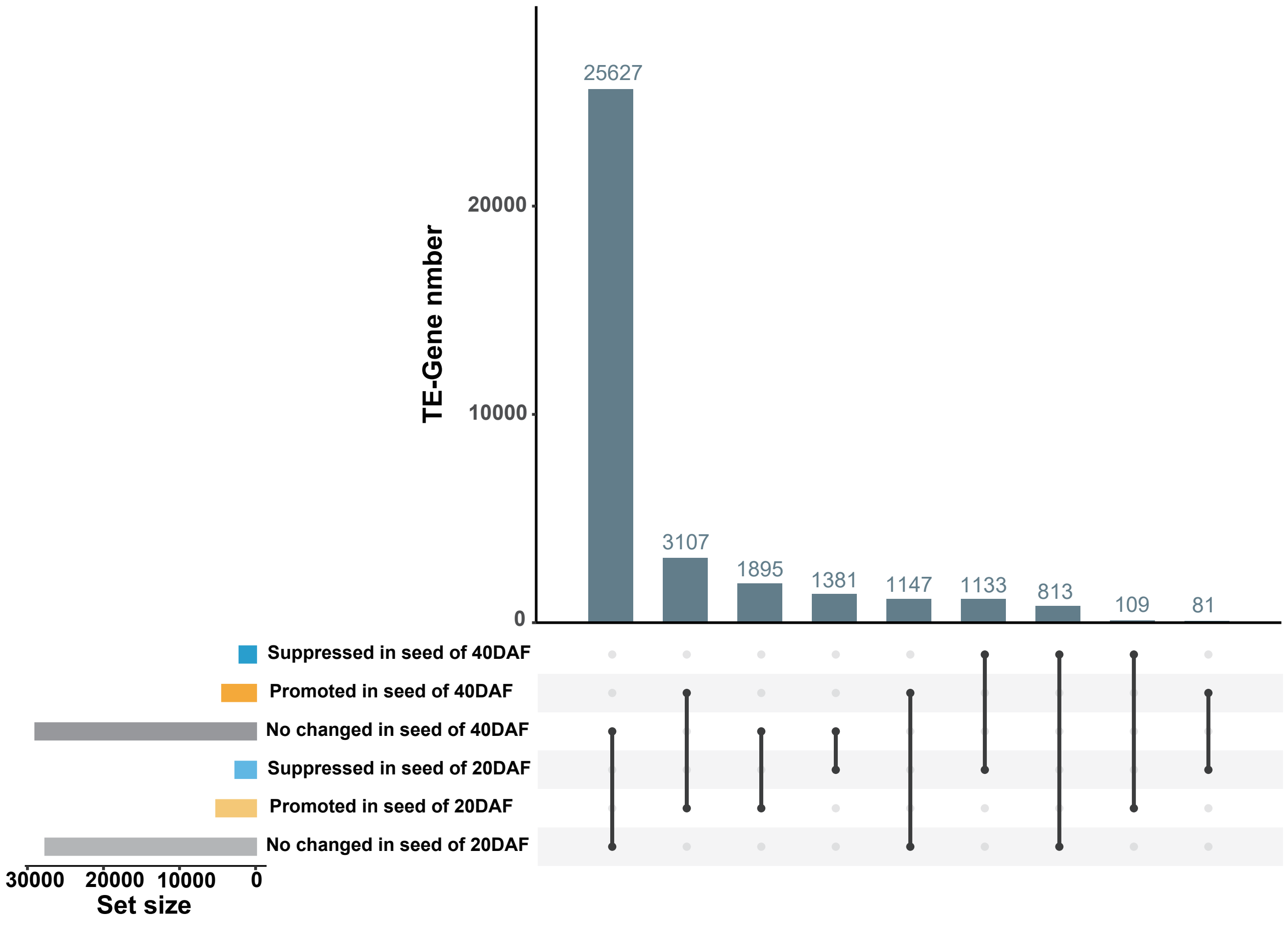

Supplementary Figure S6. Summary of relationship between TEs and adjacent gene expression levels. Summary of relationship between TEs and adjacent gene expression levels. Bars on the top panels represent numbers of different intersect combinations of no changed, promoted and suppressed TE-genes at different tissues. Bars on the left panels represent total numbers of no changed, promoted and suppressed TE-genes at different tissues.

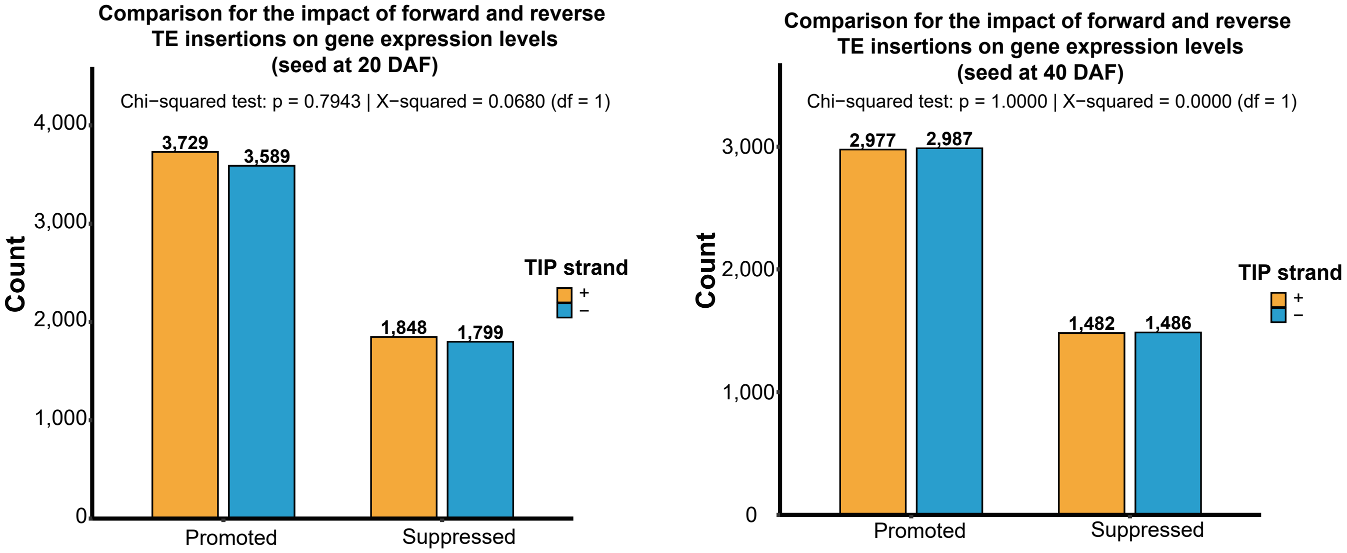

Supplementary Figure S7. Comparison for ratios of promoted and suppressed TEs with two orientations. Two panels from left to right are numbers of promoted and suppressed TEs across seeds at 20 DAF and 40DAF. Yellow and blue bars represent TEs with “+” and “-” strands, respectively.

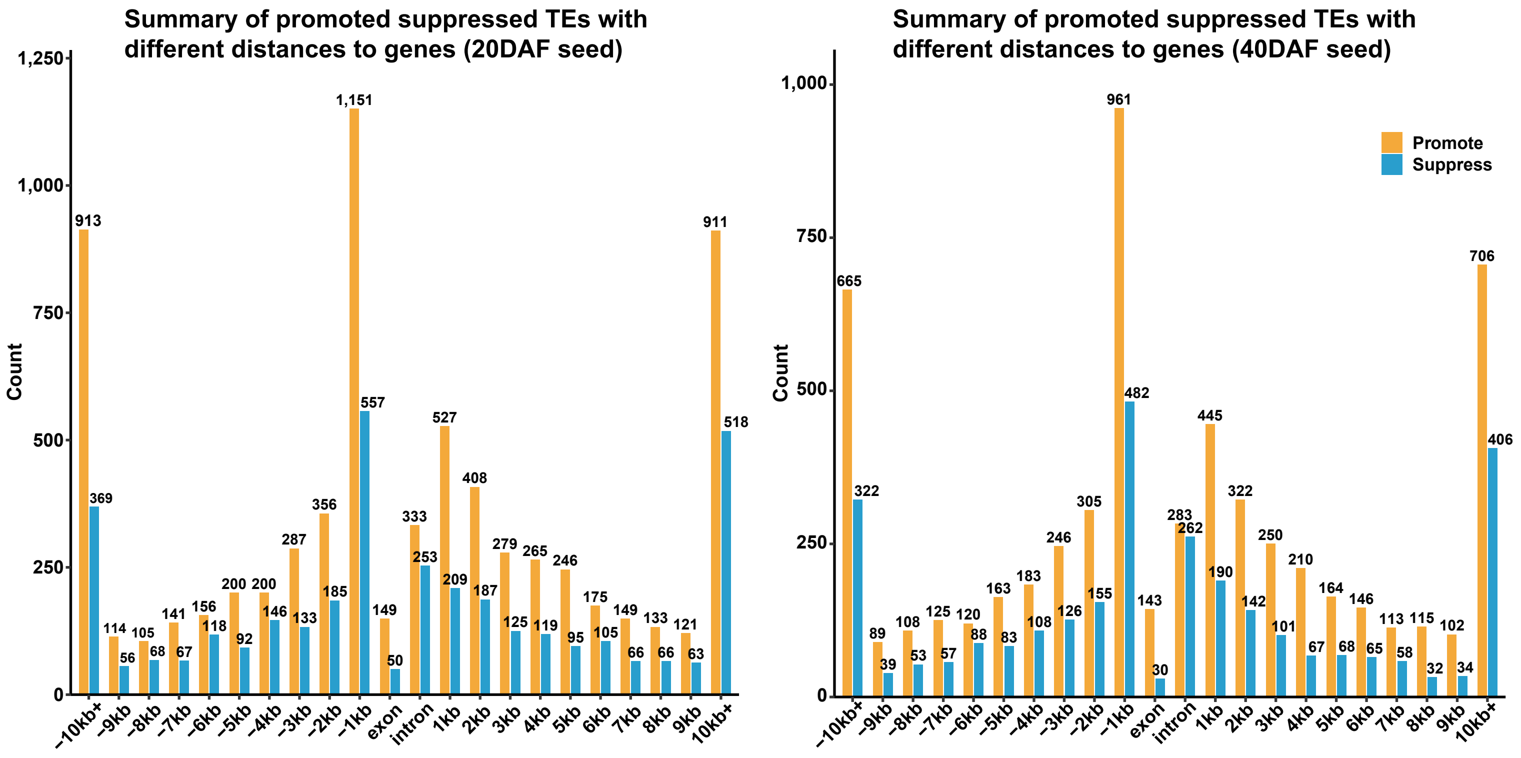

Supplementary Figure S8. Summary of promoted and suppressed TEs with different distances to genes. Left and right panels represent the RNA-seq datasets from seeds at 20 DAF and 40 DAF, respectively. Yellow and blue bars represent promoted and suppressed TEs, respectively.

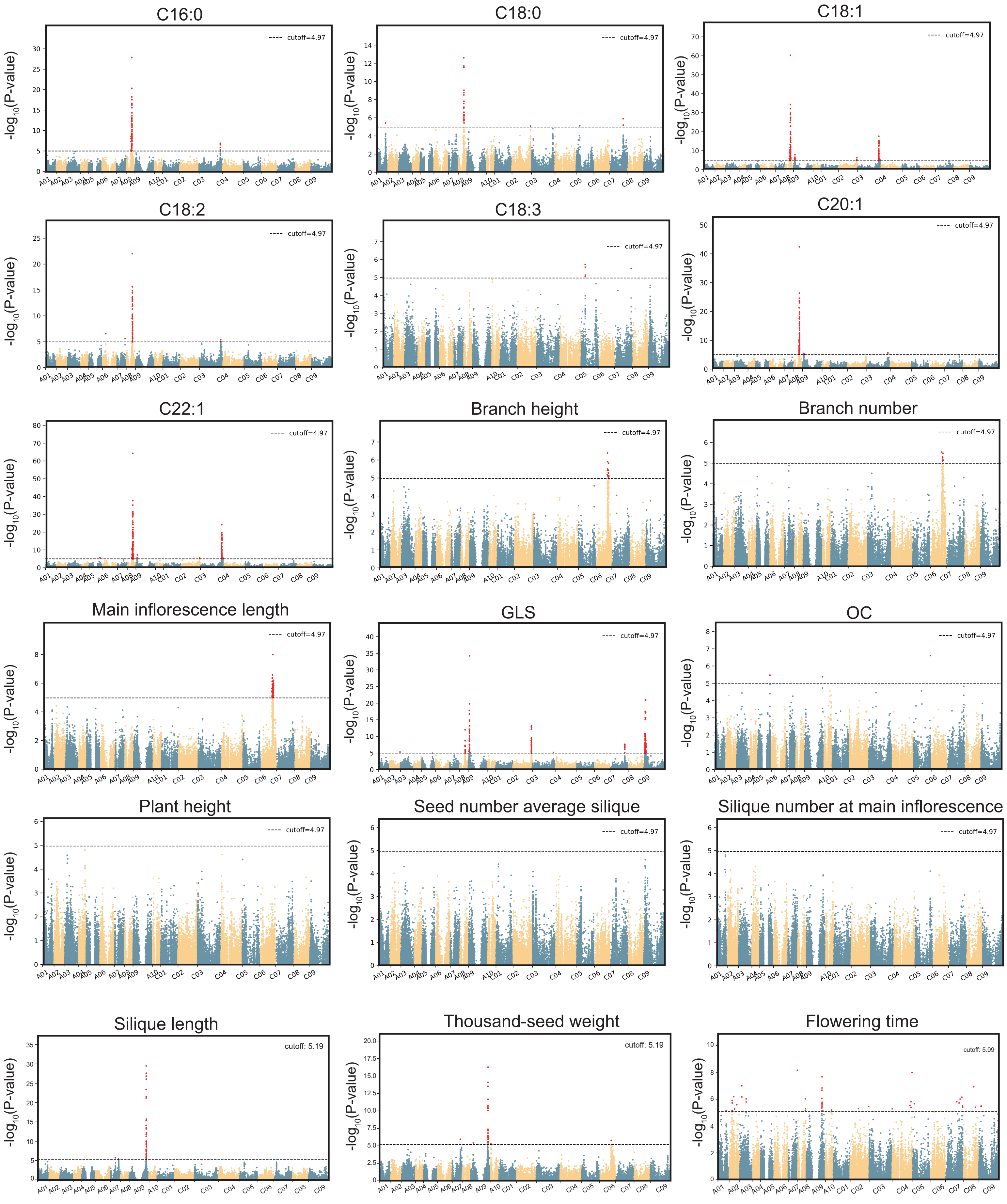

Supplementary Figure S9. Manhattan plot from GWAS of 18 phenotypes. A-B) Global manhattan plots for silique length (A) and thousand seed weight (B). Red dots represent significant TEs and the cutoff of GWAS is 5.19.

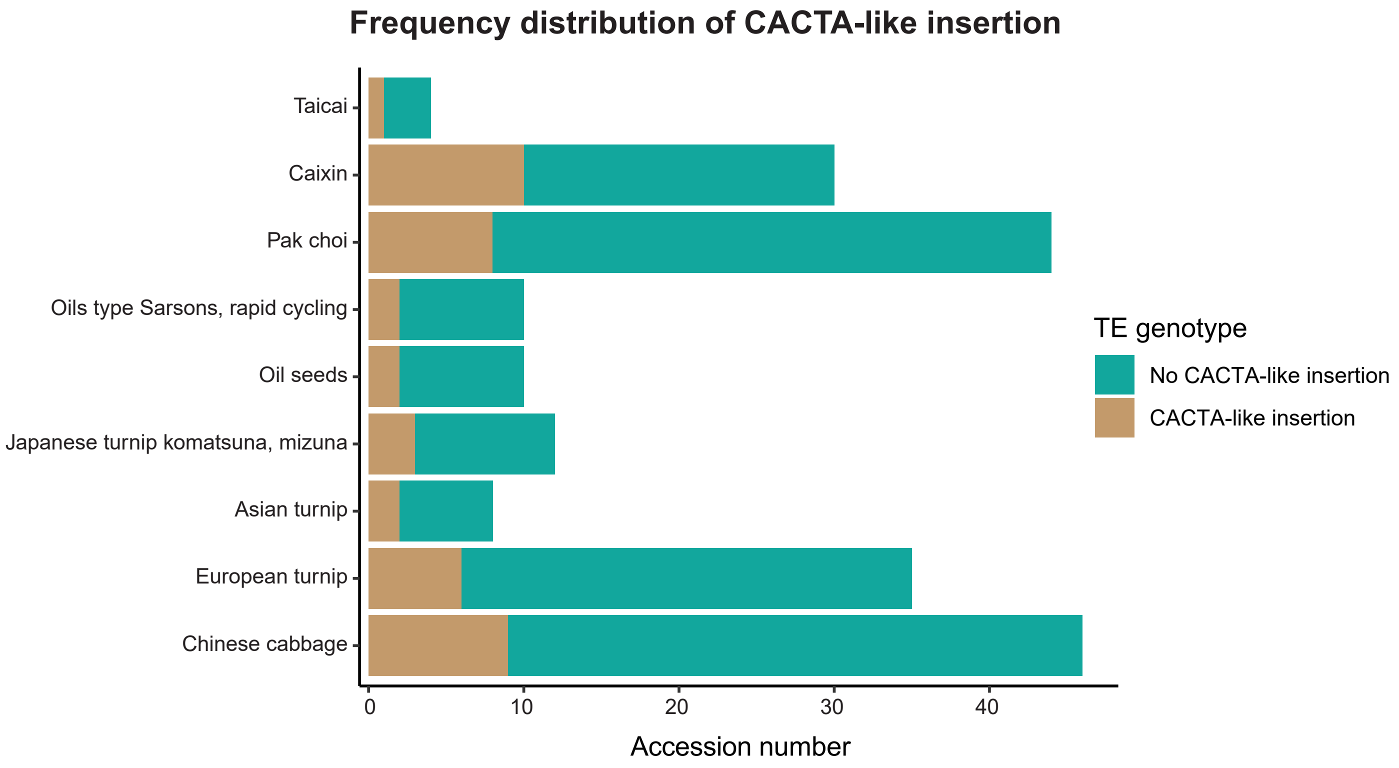

Supplementary Figure S10. Frequency distribution of CACTA-like insertion in 199 *B. rapa* accessions. Brown and green bars represent numbers of accessions with the CACTA-like insertion and no CACTA-like insertion, respectively.

A

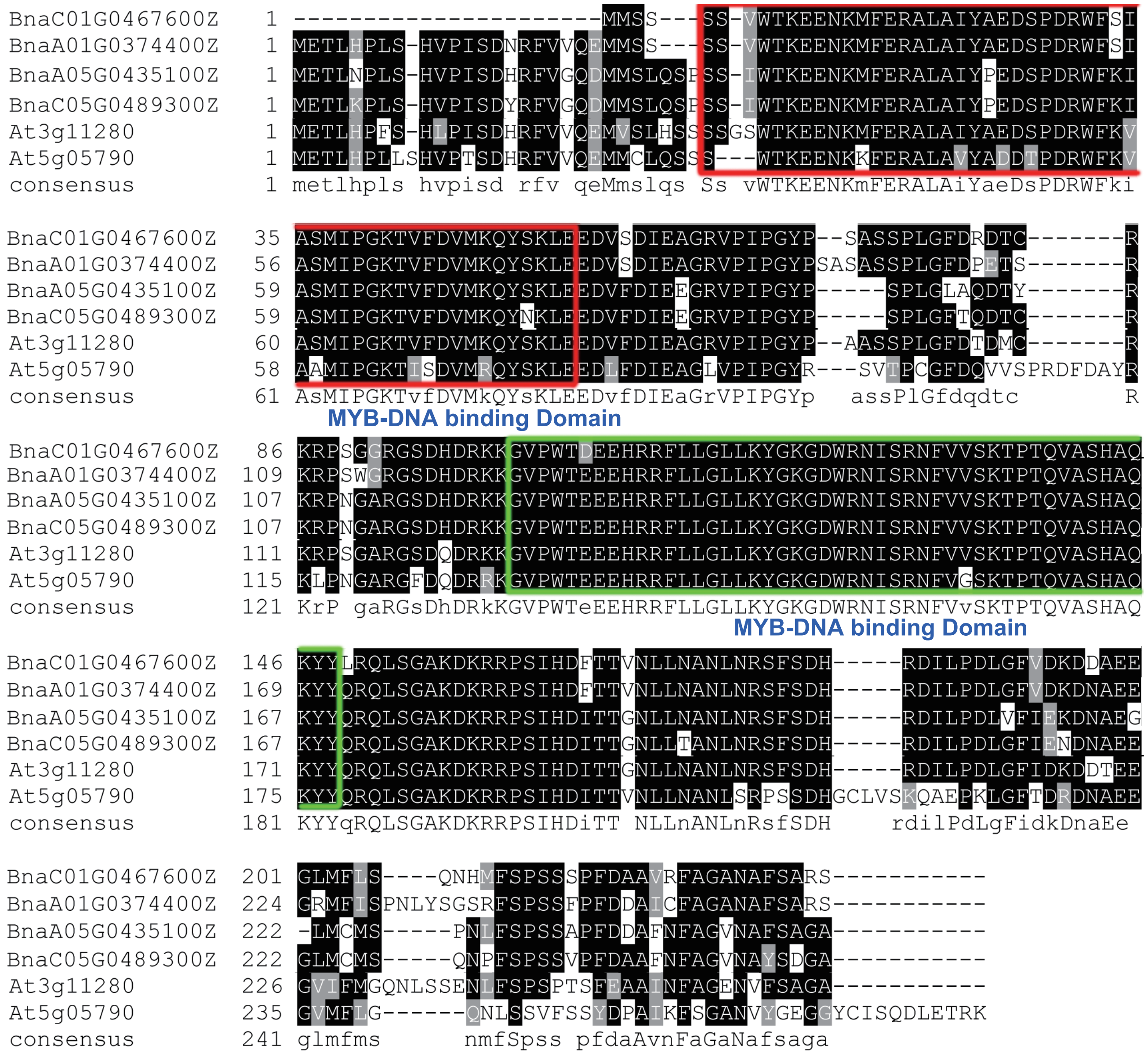

B

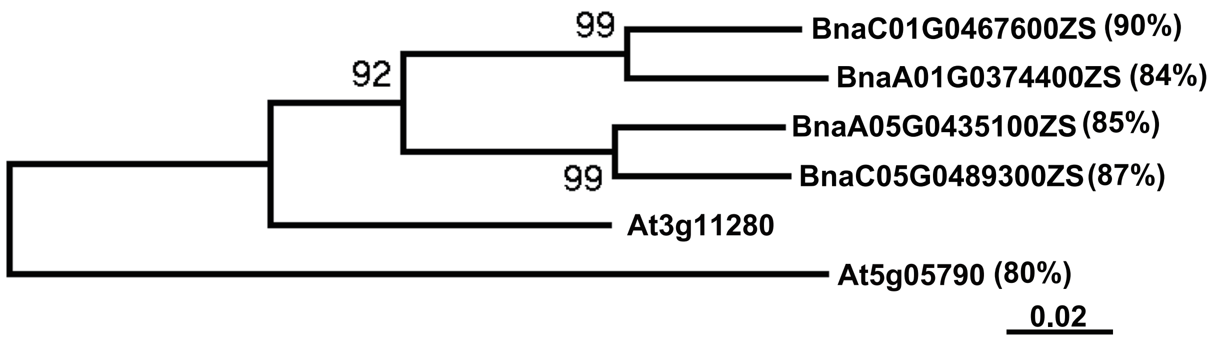

Supplementary Figure S11. Homologs of AT3G11280 and AT5G05790 in the *Brassica napus* genome. A) Multiple sequence alignment of AT3G11280, AT5G05790, BnaA01G0374400ZS, BnaA05G0435100ZS, BnaC01G0467600ZS, and BnaC05G0489300ZS. Red and green boxes indicate the MYB binding domain. B) Phylogenetic analysis of At3g11280, At5g05790 BnaA01G0374400ZS, BnaA05G0435100ZS, BnaC01G0467600ZS, and BnaC05G0489300ZS. The ratio behind the gene number is the amino acid sequence similarity of each corresponding protein compared with At3g11280.

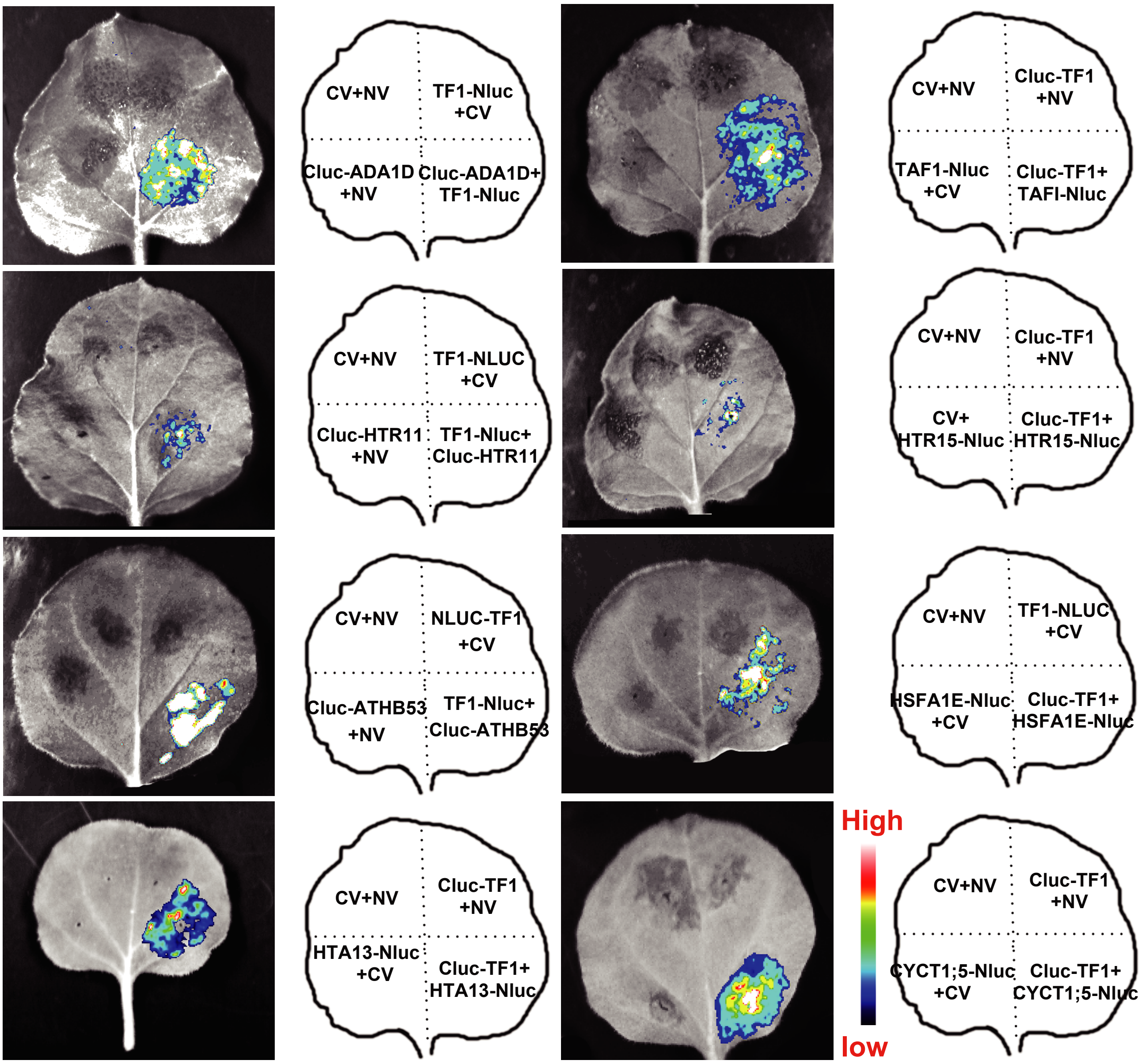

Supplementary Figure S12. Split-luciferase complementation assay confirmed BnaA01G0374400ZS interacting proteins. CV indicates C terminal fusion empty vector, NV indicates N terminal fusion empty vector, and MYB indicates BnaA01G0374400ZS.
